# NO enhances the adaptability to high-salt environments by regulating osmotic balance, antioxidant defense, and ion homeostasis in eelgrass based on transcriptome and metabolome analysis

**DOI:** 10.3389/fpls.2024.1343154

**Published:** 2024-02-07

**Authors:** Xianyan Wang, Tongtong Wang, Pei Yu, Yuchun Li, Xinfang Lv

**Affiliations:** ^1^ Marine College, Shandong University, Weihai, China; ^2^ Shandong University-Australian National University (SDU-ANU) Joint Science College, Shandong University, Weihai, China

**Keywords:** transcriptome, metabolome, salt tolerance, *Zostera marina*, NO

## Abstract

**Introduction:**

Eelgrass is a typical marine angiosperm that exhibits strong adaptability to high-salt environments. Previous studies have shown that various growth and physiological indicators were significantly affected after the nitrate reductase (NR) pathway for nitric oxide (NO) synthesis in eelgrass was blocked.

**Methods:**

To analyze the molecular mechanism of NO on the adaptability to high-salt environment in eelgrass, we treated eelgrass with artificial seawater (control group) and artificial seawater with 1 mM/L Na_2_WO_4_ (experimental group). Based on transcriptomics and metabolomics, we explored the molecular mechanism of NO affecting the salt tolerance of eelgrass.

**Results:**

We obtained 326, 368, and 859 differentially expressed genes (DEGs) by transcriptome sequencing in eelgrass roots, stems, and leaves, respectively. Meanwhile, we obtained 63, 52, and 36 differentially accumulated metabolites (DAMs) by metabolomics in roots, stems, and leaves, respectively. Finally, through the combined analysis of transcriptome and metabolome, we found that the NO regulatory mechanism of roots and leaves of eelgrass is similar to that of terrestrial plants, while the regulatory mechanism of stems has similar and unique features.

**Discussion:**

NO in eelgrass roots regulates osmotic balance and antioxidant defense by affecting genes in transmembrane transport and jasmonic acid-related pathways to improve the adaptability of eelgrass to high-salt environments. NO in eelgrass leaves regulates the downstream antioxidant defense system by affecting the signal transduction of plant hormones. NO in the stems of eelgrass regulates ion homeostasis by affecting genes related to ion homeostasis to enhance the adaptability of eelgrass to high-salt environments. Differently, after the NO synthesis was inhibited, the glyoxylate and dicarboxylate metabolism, as well as the tricarboxylic acid (TCA) cycle, was regulated by glucose metabolism as a complementary effect to cope with the high-salt environment in the stems of eelgrass. These are studies on the regulatory mechanism of NO in eelgrass, providing a theoretical basis for the study of the salt tolerance mechanism of marine plants and the improvement of terrestrial crop traits. The key genes discovered in this study can be applied to increase salt tolerance in terrestrial crops through cloning and molecular breeding methods in the future.

## 1 Introduction

Compared with freshwater angiosperms, marine angiosperms (seagrasses) occupy a unique and high-salinity marine ecological habitat. The latter evolved from the former (Les et al., 1997) and gradually adapted to the high-salinity seawater environment during its evolution (Duarte et al., 1995; Anton et al., 2018). *Zostera marina* (eelgrass) is a typical representative species of perennial seagrass. Seagrass bed is an integral part of the global marine ecosystem and plays an essential role in maintaining and stabilizing the marine ecosystem and even the global ecosystem (Lefebvre et al., 2010). As a representative higher plant that enters the sea multiple times in the evolutionary process, eelgrass is an ideal model to clarify the molecular mechanism of salt tolerance of marine higher plants. The study of its salt tolerance mechanism will have important practical significance for the development of marine agriculture and the cultivation of land salt-tolerant crop varieties in the future.

The consistency and uniqueness of the salt tolerance mechanism between eelgrass and other plants, especially terrestrial plants, are important scientific issues. So far, research on the salt tolerance mechanism of plants mainly focused on terrestrial plants, while research on marine angiosperms has been very limited. Liu et al. (2010) used the SMART (switching mechanism at the 5′ end of RNA transcript) method to construct a full-length cDNA library of eelgrass under different salinity and discovered several genes that may be related to salt tolerance. Kong et al. (2014) explored the transcriptome characteristics of eelgrass under environmental stress by high-throughput sequencing analysis, providing a theoretical basis for exploring the molecular mechanism of salt tolerance. However, due to the defects of the comparison database, there were still a large number of new genes with unknown functions in the above results, and there was no molecular and physiological experimental evidence to confirm that these genes or metabolic pathways were involved in the adaptation of eelgrass to high-salt environments. Based on genomic data, Olsen et al. (2016) found that seagrass lost genes related to ethylene, terpenoid biosynthesis, anti-ultraviolet damage and stomatal differentiation during evolution, and evolved genes related to high-salinity adaptation. On this basis, Lv et al. (2018) observed that nitrate reductase (NR) and nitrite reductase (NiR) were upregulated in eelgrass under high-salt environments, and NR-dependent NO synthesis played an essential role in the adaptation of eelgrass to high-salt environments (Lv et al., 2022; Lv et al., 2023). Therefore, it is necessary to study the molecular mechanism of NO involved in regulating the adaptation of eelgrass to marine high-salt environments.

NO is a small-molecule signal, which is widely found in various animals and plants. It not only can regulate almost all growth and development processes in plants, such as seed germination, flowering, and senescence (Durner and Klessig, 1999; Neill et al., 2002), but also can play an important role in plant response to various biotic and abiotic stresses (Molassiotis et al., 2010). NR-dependent pathway is one of the pathways for NO synthesis in plants (Hasanuzzaman et al., 2018). In general, NR is a key enzyme in the process of nitrogen metabolism, reducing NO_3_^−^ to NO_2_^−^. Under special conditions such as hypoxia, NR can also reduce NO_2_^−^ to NO (Wany et al., 2020). Lv et al. (2023) showed that the synthesis of NO in eelgrass mainly depended on the NR pathway, and the addition of an NR inhibitor, sodium tungstate (Na_2_WO_4_), significantly inhibited the formation of NO. It can be seen that, unlike terrestrial plants, NR in marine eelgrass efficiently catalyzes the synthesis of NO to participate in the adaptation of plants to the marine environment. In our previous study, we concluded that the NO produced by the NR pathway affected the amino acid metabolism and signal transduction of eelgrass by transcriptome analysis in leaves (Lv et al., 2023), which laid a solid foundation for further analysis of its molecular mechanism. Up to now, there are few studies on the molecular mechanisms of eelgrass adaptation to high-salt environments, and NO molecule provides an ideal breakthrough point. How does NO produced by the NR pathway affect the high-salt adaptability of different tissues (roots, stems, and leaves) of eelgrass? Which genes and metabolic pathways do NO affect eelgrass adaptation to high-salt environments? This is exactly the scientific question that this study aims to solve.

This study focused on how the NO produced by the NR pathway affects the high-salt adaptability of eelgrass. In order to further explore the molecular mechanism of the NO synthesis pathway affecting the salt tolerance of eelgrass, we conducted a combined analysis of transcriptomics and metabolomics. RNA-seq was used for transcriptome analysis, and liquid chromatography–tandem mass spectrometry (LC-MS/MS) was used for metabolome analysis to find differentially expressed genes (DEGs), differentially accumulated metabolites (DAMs), and significant metabolic pathways affected by NO. In this study, the related genes and metabolites of different tissues of eelgrass affected by the NO synthesis pathway were discussed from the perspective of omics combination, and the regulation map of related metabolic pathways was drawn, which provided a basis for the study of salt tolerance mechanism of other marine plants and the improvement of traits of terrestrial crops in the future.

## 2 Materials and methods

### 2.1 Eelgrass growth conditions

Eelgrass plants in this study were harvested in May 2022 and May 2023 in Shuangdao Bay (37°28′N, 121°58′E) located in the southern part of the Bohai Sea. Eelgrass plants of similar size were collected, transported to the culture chamber in sample boxes containing seawater, cleaned with deionized water, and then domesticated in an incubator containing artificial seawater for 72 h. The eelgrass plants were divided into two groups: one group was treated with artificial seawater (control group (CK group)), and the other group was treated with 1 mM/L Na_2_WO_4_ in artificial seawater (experimental group (EG group)) for 24 h. Three biological replicates were set up for each group of experiments. During domestication and culture, eelgrass plants were kept in natural light with a 14-h-light/10-h-dark photoperiod and temperature of 22°C during the day and 18°C during the night. Whole root, stem, and leaf tissues were collected after 24 h of treatment and stored at −80°C until RNA and metabolites were extracted.

### 2.2 Transcriptome sequencing

After 24 h of treatment under the above conditions, different tissues of each group of eelgrass were frozen in liquid nitrogen and stored at −80°C, cryopreserved using dry ice, and sent to Qingdao Ouyi Biotechnology Co., Ltd. (Qingdao, China) for transcriptome sequencing. Total RNA was extracted from different tissues of eelgrass using TRIzol reagent (Invitrogen, Shanghai, China). RNA purity and quantification were evaluated using the NanoDrop 2000 spectrophotometer (Thermo Scientific, Waltham, MA, USA). RNA integrity was assessed using the Agilent 2100 Bioanalyzer (Agilent Technologies, Santa Clara, CA, USA), and the transcriptome libraries were constructed using the VAHTS Universal V6 RNA-seq Library Prep Kit. The libraries were sequenced on an Illumina NovaSeq 6000 platform, and 150-bp paired-end reads were generated after passing the quality inspection of that library using Agilent 2100 Bioanalyzer. Approximately 40–53 M raw reads for each sample were obtained. Raw reads of fastq format were first processed using fastp1, and the low-quality reads were removed to obtain the clean reads. Based on the *Z. marina* genome sequence (ftp://ftp.ncbi.nlm.nih.gov/genomes/all/GCA_001185155.1_Zosma_marina.v.2.1), HISAT2 was used to perform sequence alignment between clean reads and the designated reference genome to obtain position information on the reference genome or gene and sequence characteristic information unique to the sequenced sample. The sequencing data were stored in the Sequence Read Archive (SRA) database (accession number SRR26157017–SRR26157034) of the National Center for Biotechnology Information (NCBI). A differential expression analysis was performed following our previously described approach (Lv et al., 2023). Briefly, the fragments per kilobase per million reads (FPKM) method was used for calculating the unigene expression abundance. DEG identification was performed using DESeq2 software; *q* < 0.05 and |log_2_(FC)| ≥ 1 were set as the threshold. Principal component analysis (PCA), Gene Ontology (GO) term enrichment analysis, and Kyoto Encyclopedia of Genes and Genomes (KEGG) analysis of DEGs were performed using R (v 3.2.0).

### 2.3 Quantitative RT-PCR

Primers were designed using NCBI (https://www.ncbi.nlm.nih.gov/) (**Supplementary Table 1**). RNA-seq analysis was performed following the protocol of Lv et al. (2018). Total RNA was extracted using TransZol reagent (TransGen, Beijing, China). The first-strand cDNA was synthesized using TransScript Uni All in One First Strand cDNA Synthesis SuperMix for qPCR kit, and qRT-PCR was performed using the ABI QuantStudio 1 qRT-PCR system and PerfectStart Green qPCR SuperMix kit. The qRT-PCR analysis of each gene was performed in three biological replicates with three technical repeats per experiment. The amplification program consisted of one cycle at 94°C for 30 s, followed by 45 cycles at 94°C for 5 s, and finally 45 cycles at 60°C for 30 s. After the reaction, the specificity of the reaction was verified by melting curve analysis. β-Actin (Zosma01g00180) was used as the internal reference gene, and the 2^−ΔΔCt^ method was used to calculate the relative change of relative gene expression.

### 2.4 Metabolome profiling analysis

The metabolome analysis material was the same as the transcriptome analysis material, with three biological replicates per treatment group. Metabolite extraction and LC-MS analysis were performed by Qingdao Ouyi Biotechnology Co., Ltd. (Qingdao, China). A sample at a volume of 80 mg was transferred to a 1.5-mL tube and abstracted with 1 mL cold methanol–water and 20 μL internal standard (L-2-chlorophenylalanine, 0.06 mg/mL; methanol configuration) after being stored at −20°C for 2 min and then grounded at 60 Hz for 2 min. The sample was ultrasonically extracted in an ice water bath for 30 min and stored overnight at −20°C. After 10-min centrifugation (13,000 rpm and 4°C), the supernatant was filtered and transferred to LC injection vials and stored at −80°C until LC-MS analysis. LC-MS/MS analysis was performed using a liquid chromatography–mass spectrometer (AB Sciex, Framingham, MA, USA) consisting of AB ExionLC ultra-high-performance liquid phase tandem QE plus a high-resolution mass spectrometer. The chromatography was performed on an ACQUITY UPLC HSS T3 (100 mm × 2.1 mm, 1.8 um) column at 45°C. The mobile phase was water (containing 0.1% formic acid) and acetonitrile (containing 0.1% formic acid), the flow rate was 0.35 mL/min, and the injection volume was 2 μL. Electrospray ionization (ESI) ion source was used for mass spectrometry, and positive and negative ion scanning modes were used to collect signals. Qualitative and relative quantification analyses of raw data were performed using the metabolome data processing software Progenesis QI v2.3 (Nonlinear Dynamics, Newcastle, UK), and standardization pre-processing of raw data was carried out. Multivariate statistical analysis was performed using PCA to distinguish the overall difference in metabolic profiles between groups. Variable importance in projection (VIP) > 1 and *p* < 0.05 were set as the threshold to screen out differential metabolites between samples.

### 2.5 Verification of DAM content

The contents of ascorbic acid, allantoic acid, curcumin, proline, and chlorogenic acid in eelgrass were determined by spectrophotometry with a UV752N ultraviolet–visible spectrophotometer (YoKe, Shanghai, China). The content of lysine was determined by high-performance liquid chromatography (HPLC) using an Agilent 1200 HPLC liquid chromatograph (Agilent Technologies, California). Three biological replicates were performed in each group.

The ascorbic acid concentration was determined using the methods described by Maliya et al. (2019). Root samples (1.25 g) were ground in a chilled mortar and homogenized in 25 mL of 1% hydrochloric acid solution. The mixture was centrifuged at 13,000 rpm/min for 4 min, and the supernatant was filtered. The samples were measured at 243 nm using a 1% hydrochloric acid solution for a blank.

The content of allantoic acid was determined after modification according to the method of Brychkova et al. (2008). The root samples (200 mg) were extracted in 0.8 mL of 80% ethanol. After centrifugation at 13,000 r/min for 5 min at 4°C, 300 μL H_2_O and 100 μL of 0.15 mol/L hydrochloric acid were added to 100 μL of extraction solution and underwent 100°C constant temperature water bath for 8 min and then ice bath for 4 min. At room temperature, 100 μL of 0.4 mol/L phosphate buffer and 100 μL of 23.0 mM/L phenylhydrazine hydrochloride were added. The homogenate was mixed with 500 μL concentrated hydrochloric acid and 100 μL of 50.0 mM/L potassium ferricyanide. The absorbance was measured at the wavelength of 535 nm after centrifugation for 5 min at 10,000 rpm/min (4°C).

The curcumin content was quantified using visible spectrophotometry as described by Tang et al. (2002). The stem tissue of eelgrass was dried at 60°C for 8 h and ground using a mortar. The stem powders (1 g) were mixed with 25 mL of methanol and shaken for 5 h. The mixture was centrifuged at 13,000 rpm/min for 5 min, the supernatant was transferred to a new test tube, and the absorbance was read at 420 nm.

The proline content was estimated using the standard procedure described by Lv et al. (2023). The samples (500 mg) were extracted in 5 mL of 3% of sulfosalicylic acid. The homogenate was centrifuged, and 2 mL of supernatant was treated with a mixture of 2 mL glacial acetic acid and 3 mL acid ninhydrin by boiling at 100°C for 40 min. After cooling, 4 mL of toluene was added. The upper liquid was centrifuged at 3,000 rpm/min for 5 min. The chromophore containing toluene was transferred to a new test tube, and the absorbance at 520 nm was read using toluene as a blank.

Chlorogenic acid was determined using the methods described by Choma et al. (2019). The control and treated samples (1 g) were triturated in 50 mL of 95% ethanol and placed in a constant temperature water bath at 85°C for 4 h. After cooling the mixture to room temperature, the solution was fixed capacity to 250 mL with 95% ethanol, and then the absorbance at a wavelength of 330 nm was measured.

High-performance liquid chromatography as described by Daliri et al. (2021) was used to determine the lysine content. Leaf samples (100 mg) were ground in a chilled mortar and mixed with 5 mL of 6 mol/L hydrochloric acid solution. The hydrolysis reaction was carried out at 110°C for 24 h, and then 5 mL of 6 mol/L sodium hydroxide solution was added and shaken well for 1 min. After high-speed centrifugation at 5,000 rpm/min for 10 min, 0.5 mL supernatant was added with 0.5 mL of 0.5 mol/L sodium bicarbonate solution with a pH of 9.0 and 0.5 mL of DNFB solution. After 60 min of constant temperature water bath at 60°C, the mixture was cooled to room temperature. The solution was fixed capacity to 5 mL with phosphate buffer (pH = 7.0) and vortexed for 1 min. After allowing it to stand in the dark for 15 min, 1-mL solution was taken and filtered through the membrane (0.22 μm). The samples were passed through a C18 chromatographic column (250 × 4.6 mm, 5 µm) at 40°C, and the mobile phase contained l mol/L sodium acetate solution (pH = 5.3) and methanol (containing water of the same volume). The flow rate was held at 1 mL/min, and the injection volume was 20 μL.

### 2.6 Conjoint analysis of transcriptome and metabolome

By simultaneously mapping DEGs and DAMs to the KEGG Pathway database, their common pathway information was obtained. Spearman’s correlation coefficient (Cor) and *p*-value were calculated using the Cor function of R language. Finally, a network map of DEGs-DAMs based on Spearman’s correlation coefficient (with |Cor| > 0.8 and *p* ≤ 0.05) using Cytoscape was constructed.

### 2.7 Statistical analysis

In the experiment of qRT-PCR and content verification of DAMs, the statistical analysis was performed using SPSS software, *p* ≤ 0.05 was considered to be significantly different in the t-test, and the final graph was plotted using Origin software.

## 3 Results

### 3.1 Overview of transcriptomic responses of eelgrass treated with Na_2_WO_4_

To obtain genetic information on the salinity tolerance of eelgrass affected by the NO synthesis pathway, all transcript information in different tissues (roots, stems, and leaves) of the CK group and EG group was identified by RNA-seq analysis. A total of 41.84–52.73 million raw reads were obtained from samples of the CK group and EG group. A total of 39.5–50.1 million clean reads were obtained. The proportions of Q30 were 91.38–94.47%, and the GC content of clean reads was 44.57–46.98%, indicating that the transcriptome sequencing data were of high quality. After a trimming process in the low-quality regions and adapter sequences were removed, the clean reads were mapped in the reference genome sequences of eelgrass with the mapping ratio of 93.32%–99.97% and the uniquely mapped reads of 83.26%–93.73% (**Table 1**).

**Table 1:** Transcriptome sequencing results statistics.

Sample	Raw reads (M)	Clean reads (M)	Q30 (%)	GC (%)	Total mapped	Uniquely mapped
CK1LZ	52.73	50.08	91.94	46.38	47,340,634 (94.54%)	42,729,786 (85.33%)
CK1RZ	44.08	43.76	94.21	45.04	43,750,869 (99.97%)	40,954,951 (93.58%)
CK1SZ	50.93	50.1	92.01	45.26	47,533,305 (94.87%)	44,907,397 (89.63%)
CK2LZ	50.67	48.14	91.56	46.47	45,280,572 (94.06%)	40,080,511 (83.26%)
CK2RZ	45.73	45.44	94.47	44.57	45,424,522 (99.97%)	42,696,457 (93.97%)
CK2SZ	47.67	46.55	92	44.67	43,440,972 (93.32%)	41,137,319 (88.37%)
CK3LZ	49.93	48.77	91.68	46.62	45,732,625 (93.77%)	41,265,628 (84.61%)
CK3RZ	43.36	43.07	94.01	45	43,047,394 (99.94%)	40,183,828 (93.29%)
CK3SZ	40.68	39.5	91.52	45.24	37,228,427 (94.25%)	35,355,319 (89.51%)
EG1LZ	47.73	46.88	92.19	46.5	44,906,971 (95.80%)	40,416,514 (86.22%)
EG1RZ	41.84	41.55	93.71	44.89	41,532,489 (99.97%)	38,941,801 (93.73%)
EG1SZ	49.4	48.61	91.94	45.26	46,521,100 (95.70%)	44,030,521 (90.57%)
EG2LZ	48.93	47.53	91.81	46.98	45,042,204 (94.76%)	40,374,171 (84.94%)
EG2RZ	44.83	44.53	94.19	44.98	44,515,074 (99.97%)	41,651,505 (93.54%)
EG2SZ	47.07	45.8	91.4	45.39	43,251,681 (94.43%)	40,741,949 (88.95%)
EG3LZ	48.07	46.64	91.62	46.01	44,605,705 (95.65%)	40,598,685 (87.05%)
EG3RZ	43.2	42.92	94.18	44.9	42,909,347 (99.97%)	40,250,390 (93.78%)
EG3SZ	46.4	45.91	91.38	45.07	43,579,166 (94.92%)	41,016,479 (89.34%)

**Figure 1:**
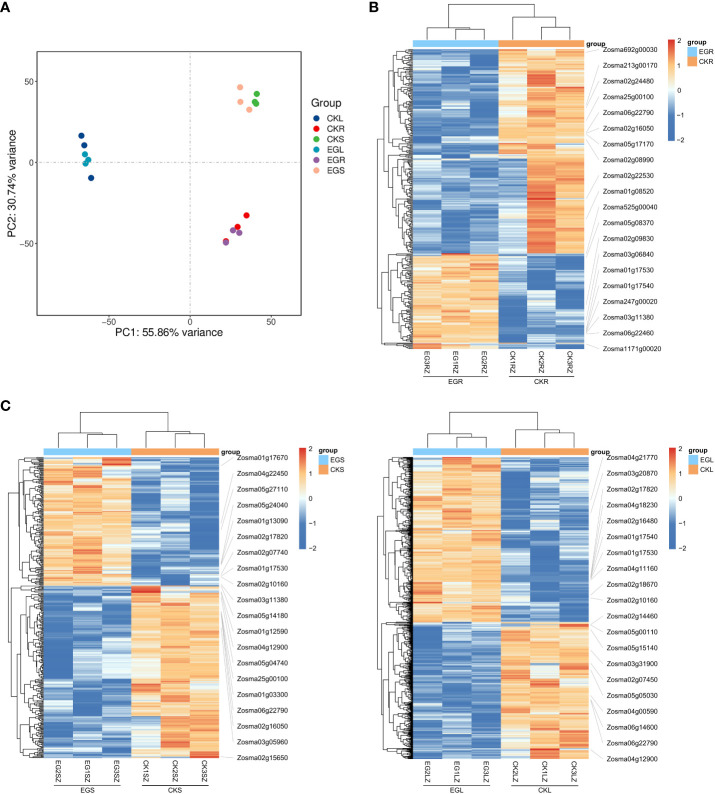
The transcriptomic analysis in eelgrass treated with Na_2_WO_4_. **(A)** PCA clustering based on transcriptome data. Heat map of DEGs in roots **(B)**, stems **(C)**, and leaves **(D)**. PCA, principal component analysis; DEGs, differentially expressed genes.

The PCA results showed a clear separation between the different tissues by PC1 (**Figure 1A**). A clustering analysis of expression patterns of genes with significant differences in expression between two comparative groups was conducted. Through heat maps (**Figures 1B-D**), it can also be visually observed that the three biological replicates have similar trends, and there were significant obvious differences between the CK group and EG group. Based on a threshold of *q* < 0.05 and |log_2_ (FC)| ≥ 1, a total of 326 (103 upregulated and 223 downregulated), 368 (157 upregulated and 211 downregulated), and 859 (470 upregulated and 389 downregulated) DEGs were detected in the CKR *vs.* EGR, CKS *vs.* EGS, and CKL *vs.* EGL, respectively (**Supplementary Figure 1**). These results indicated that inhibition of NO synthesis affected the gene expression levels in all tissues of eelgrass under high-salt environments.

### 3.2 The functional analysis of DEGs involved in NO regulation under high salinity in eelgrass

To explore the function of these DEGs and the biological pathways associated with the DEGs, we performed GO term enrichment analysis and KEGG analysis in different tissues.

**Figure 2:**
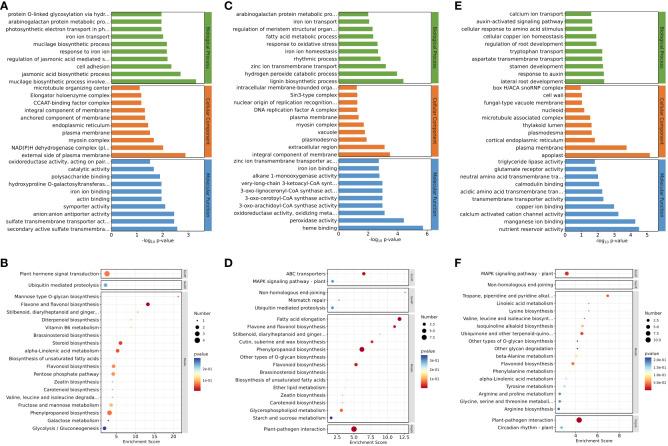
Down-regulated DEGs enriched on GO terms and KEGG pathways after inhibition of NO synthesis. Top 30 GO terms of DEGs in roots **(A)**, stems **(C)**, and leaves **(E)**. Significantly enriched KEGG pathways of DEGs in roots **(B)**, stems **(D)**, and leaves **(F)**. DEGs, differentially expressed genes; GO, Gene Ontology; KEGG, Kyoto Encyclopedia of Genes and Genomes.

For downregulated genes in roots after inhibition of NO synthesis, the significantly enriched GO terms were “jasmonic acid biosynthetic process” (*p* = 1.98E−3), “regulation of jasmonic acid mediated signaling pathway” (*p* = 6.41E−03), “secondary active sulfate transmembrane transporter activity” (*p* = 2.68E−03), “sulfate transmembrane transporter activity” (*p* = 3.55E−03), and “anion–anion antiporter activity” (*p* = 3.55E−03) (**Figure 2A**). These results suggested that jasmonate biosynthesis and its signaling pathways were inhibited in eelgrass after the NO synthesis pathway was inhibited, and genes related to ion transmembrane transporter activity were also affected, all of which play important roles in high-salt adaptation. KEGG enrichment analysis showed that phenylpropanoid biosynthesis, flavonoid biosynthesis, flavone, and flavonol biosynthesis were significantly enriched in the downregulated genes (**Figure 2B**), indicating that the inhibition of NO synthesis also had a notable influence on the biosynthesis of flavonoids in eelgrass. **Supplementary Table 2** lists several genes in the significant enrichment pathways. Compared to the CK group, the expression levels of genes involved in the synthesis of phenylpropanoid and flavonoid in the EG group, such as 3-ketoacyl-CoA thiolase (KAT2) (Zosma06g19680), 4-coumarate-CoA ligase like (4CL) (Zosma06g25690), and allene oxide synthase (AOS) (Zosma01g01290) were downregulated by 0.284-, 0.426-, and 0.410-fold, respectively.

For downregulated genes in stems after inhibition of NO synthesis, the significantly enriched GO terms were “zinc ion transmembrane transport” (*p* = 6.35E−04), “iron ion homeostasis” (*p* = 2.15E−03), “iron ion transport” (*p* = 8.46E−03), “oxidoreductase activity, oxidizing metal ions” (*p* = 7.65E−04), “zinc ion transmembrane transporter activity” (*p* = 1.90E−03), and other pathways (**Figure 2C**). KEGG enrichment analysis showed that fatty acid elongation, ABC transporters, and other pathways were significantly enriched in the downregulated genes after inhibiting NO synthesis (**Figure 2D**). These results suggested that the inhibition of NO synthesis had a significant impact on the genes involved in the biological processes related to ion transmembrane transport and ion homeostasis in eelgrass. **Supplementary Table 3** lists several genes in the significant enrichment pathways. Compared to the CK group, the expression levels of genes involved in synthesis of long-chain fatty acid or substance transport in the EG group, such as ketoacyl-CoA reductase (KCR) (Zosma02g09920), ABC transporter B family member 21 (Zosma06g17130), and zinc transporter (Zosma01g21230), were downregulated by 0.316-, 0.080-, and 0.231-fold, respectively.

For downregulated genes in leaves after inhibition of NO synthesis, the significantly enriched GO terms were “response to auxin” (*p* = 4.35E−03), “auxin-activated signaling pathway” (*p* = 2.22E−02), and “calmodulin binding” (*p* = 8.37E−03) (**Figure 2E**). These results indicated that the inhibition of NO synthesis affected the biosynthesis of auxin and the calmodulin binding in eelgrass. KEGG enrichment analysis showed that MAPK signaling pathway-plant, tropane piperidine, and pyridine alkaloid biosynthesis were significantly enriched in the downregulated genes after inhibiting NO synthesis (**Figure 2F**). This showed that signal transduction was related to the NO synthesis pathway and played an important role in the adaptation to high-salt adaptation in eelgrass. **Supplementary Table 4** lists several genes in the significant enrichment pathways. Compared to the CK group, the expression levels of genes related to auxin synthesis and calcium signaling in the EG group, such as auxin-responsive protein (Zosma05g13460), Centrin-2 (Zosma03g31500), and calcium-binding EF-hand family protein (Zosma03g31770) were downregulated by 0.383-, 0.300-, and 0.124-fold, respectively.

In addition to analyzing the downregulated genes, we also examined the upregulated genes. For the upregulated genes in the EG group, the most significantly enriched GO terms in roots were “naringenin-chalcone synthase activity” (*p* = 5.13E−05), “transmembrane receptor protein serine/threonine kinase activity” (*p* = 4.82E−03), and “response to wounding” (*p* = 1.50E−02) (**Supplementary Figure 2A**). KEGG enrichment analysis in roots showed that the flavonoid biosynthesis metabolic pathway was significantly enriched (**Supplementary Figure 2B**). These results showed that genes related to flavonoid biosynthesis and transmembrane transport in eelgrass were closely related to NO. GO enrichment analysis in stems showed that the metabolic pathways such as “glucose metabolic process” (*p* = 1.17E−02) and “glyceraldehyde-3-phosphate dehydrogenase (NAD^+^) (phosphorylation) activity” (*p* = 2.14E−03) were significantly enriched (**Supplementary Figure 2C**). KEGG enrichment analysis showed that linoleic acid metabolism and inositol phosphate metabolism pathways were significantly enriched in stems (**Supplementary Figure 2D**). These results indicated that genes related to energy metabolism and plant signaling in eelgrass were influenced by the NO synthesis. GO enrichment analysis in leaves of eelgrass showed that the metabolic pathways such as “response to salt stress” (*p* = 1.66E−03) and “response to wounding” (*p* = 1.48E−03) were significantly enriched (**Supplementary Figure 2E**). KEGG enrichment analysis showed that MAPK signaling pathway-plant, flavonoid biosynthesis, ubiquinone, and other terpenoid-quinone biosynthesis pathways were enriched considerably in leaves (**Supplementary Figure 2F**). These results suggested that the inhibition of the NO synthesis in eelgrass affected its pathways of response to salt stress, injury, and signal transduction.

**Figure 3:**
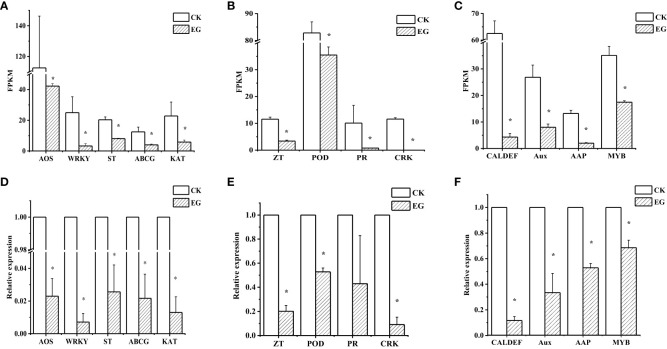
qRT-PCR validation of selected downregulated genes in different tissues. Expression patterns of selected genes based on the FPKM data in roots **(A)**, stems **(B)**, and leaves **(C)**. qRT-PCR was performed to obtain the relative expression levels in roots **(D)**, stems **(E)**, and leaves **(F)**. The error bars represent the standard deviations from three replicates. The asterisk indicates a significant difference (*p* < 0.05). The selected genes are as follows: AOS (allene oxide synthase, Zosma01g01290), WRKY (WRKY transcription factor 7, Zosma05g26860), ST (sulfate transporter, Zosma01g16740), ABCG (ABC transporter G family member 1, Zosma03g13310), KAT (3-ketoacyl-CoA thiolase, Zosma06g19680), ZT (zinc transporter, Zosma01g21240), POD (peroxidase, Zosma04g27070), PR (pathogenesis-related protein PR-4B, Zosma05g22610), CRK (cysteine-rich receptor-like protein kinase 8, Zosma05g04740), MYB (myb-like transcription factor family protein, Zosma01g07420), Aux (auxin-responsive protein, Zosma01g08410), AAP (amino acid permease 6, Zosma05g18650), CAL-DEF (calcium-binding EF-hand family protein, Zosma03g31770).

To verify the reliability of the RNA-seq data, we selected 13 downregulated genes and determined their relative expression levels in the CK group and the EG group by qRT-PCR. The experimental results showed that the expression patterns of the 13 genes were similar to those obtained by RNA-seq analysis (**Figure 3**). Similarly, we performed qRT-PCR for upregulated genes (**Supplementary Figure 3**). For each gene, the RNA-seq data were similar to qRT-PCR results, which proved the reliability of transcriptome data.

### 3.3 Metabolome analysis of eelgrass treated with Na_2_WO_4_

**Figure 4:**
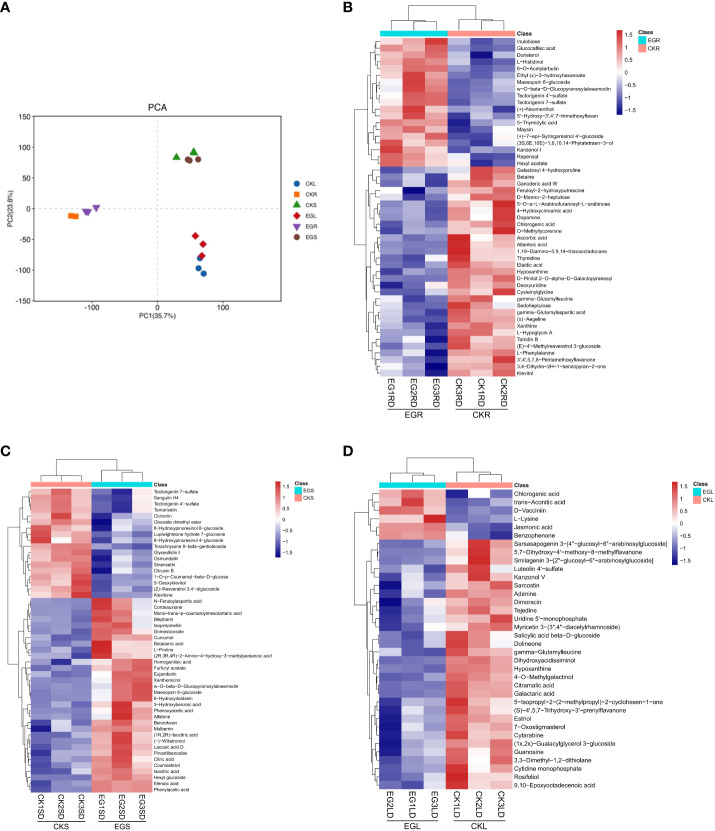
The metabolite analysis in eelgrass treated with Na_2_WO_4_. **(A)** PCA clustering based on metabolome data. Heat map of DAMs in roots **(B)**, stems **(C)**, and leaves **(D)**. PCA, principal component analysis; DAMs, differentially accumulated metabolites.

In order to obtain metabolites affected by the NO synthesis pathway in eelgrass, a widely untargeted metabolome analysis was performed using an LC-MS system. In total, 2,419, 2,382, and 2,343 metabolites were detected in roots, stems, and leaves, respectively. The PCA showed a clear separation between the different tissues by PC1 (**Figure 4A**). The heat map analysis showed that the contents of various metabolites in the replicate samples had similar trends (**Figures 4B-D**), indicating that the metabolome data were of high quality.

In this study, the changes of DAMs in different tissues of eelgrass under the CK group and EG group were detected based on non-targeted metabolome technology. Based on a threshold of VIP > 1 and *p* < 0.05, DAMs between the EG group and CK group in different tissues were screened. A total of 63 (42 downregulated and 21 upregulated), 52 (19 downregulated and 33 upregulated), and 36 (30 downregulated and 6 upregulated) DAMs were detected in the roots, stems, and leaves, respectively (**Supplementary Figure 4**). There were no common DAMs in different tissues, indicating that DAMs exhibited tissue specificity.

**Figure 5:**
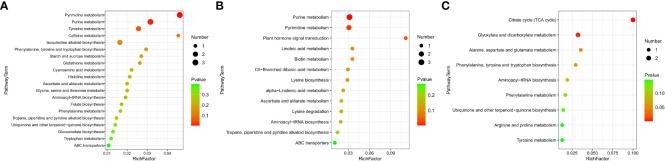
DAMs enriched on KEGG pathways after inhibition of NO synthesis. Significantly enriched KEGG pathways of differentially abundant metabolites in roots **(A)**, leaves **(B)**, and stems **(C)**. DAMs, differentially accumulated metabolites; KEGG, Kyoto Encyclopedia of Genes and Genomes.

The KEGG enrichment analysis based on DAMs showed that several KEGG pathways were significantly enriched in different tissues. This suggests that these metabolic pathways might play significant roles during NO regulation in eelgrass. DAMs in roots were enriched in purine and pyrimidine metabolic pathways (**Figure 5A**). The results showed that several metabolites were downregulated after inhibition of NO synthesis, such as various nucleotides (hypoxanthine, xanthine, deoxyuridine, and thymidine), organic acids (allantoic acid and chlorogenic acid), amino acids (l-phenylalanine and cysteinylglycine), phenylpropanoids, and polyketides (**Supplementary Table 5**). Several metabolites were upregulated after inhibition of NO synthesis, such as nucleotides (5-thymidylic acid), phenylpropanoids, polyketides (diosmetin and maysin), and other metabolites (**Supplementary Table 5**). Purine and pyrimidine metabolism belongs to basic metabolism, indicating that the basic metabolism of roots was significantly affected. They were involved in the formation of nitrogen metabolism and other metabolic intermediates, which can protect the eelgrass from high-salt environmental pressure. DAMs in leaves were enriched in purine and pyrimidine metabolic pathways and plant hormone signal transduction pathways (**Figure 5B**). The results showed that various nucleotides and organic acids were also downregulated in leaves, which is similar to the result in roots. In addition, the contents of carbohydrates and carbohydrate conjugates (salicylic acid and beta-d-glucoside), lipids, and lipid-like molecules (9,10-epoxyoctadecenoic acid) were significantly downregulated in leaves. Meanwhile, the contents of jasmonic acid, organic acids (*trans*-aconitic acid and chlorogenic acid), amino acid (l-lysine), and other metabolites were significantly upregulated (**Supplementary Table 6**). DAMs in plant hormone signal transduction pathways were also significantly enriched, and the most significantly enriched DAM was jasmonic acid. This indicated that NO not only had a great influence on the jasmonic acid synthesis in leaves but also played a role in the synthesis of other plant hormones. It was found that auxin-related pathways were also significantly enriched, indicating that plant hormones may be the most significantly affected pathway by NO in leaves.

Unlike the roots and leaves, most of the DAMs in the stems of eelgrass were upregulated. After the NO synthesis pathway in the stems of eelgrass had been inhibited, the contents of metabolites such as organic acids and other derivatives (citric acid and isocitric acid), lipids, and lipid-like molecules (hexyl glucoside and curcumol), phenylpropanoids and polyketides (mulberrin, xanthomicrol, and 6-hydroxydaidzein), amino acids, peptides and analogs (betalamic acid and l-proline), and other metabolites (homogentisic acid) were significantly upregulated (**Supplementary Table 7**). In addition, the contents of metabolites such as phenylpropanoids and polyketides (1-*O*-*p*-coumaroyl-beta-d-glucose, cichoriin, and tamarixetin) were significantly downregulated (**Supplementary Table 7**). DAMs in stems were enriched in the tricarboxylic acid cycle (TCA cycle) and glyoxylate and dicarboxylate metabolism pathways (**Figure 5C**), indicating that the energy metabolism in stems was significantly affected after the NO synthesis pathway had been inhibited, which further affected the synthesis of other related metabolites.

**Figure 6:**
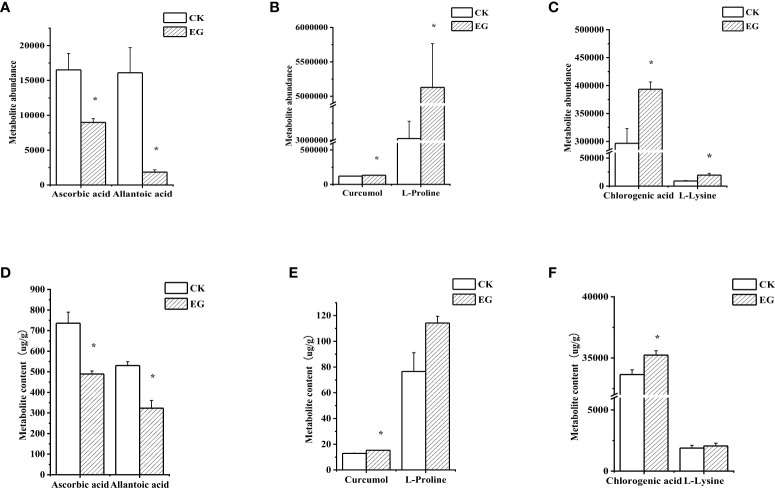
Validation of selected metabolites in different tissues. The contents of selected metabolites based on the metabolome data in roots **(A)**, stems **(B)**, and leaves **(C)**. The contents of selected metabolites based on the experimental data in roots **(D)**, stems **(E)**, and leaves **(F)**. The error bars represent the standard deviations from three replicates. The asterisk indicates a significant difference (*p* < 0.05).

To verify the reliability of metabolome data, we detected six DAMs using extra methods (**Figure 6**). After measurement, the changes in the contents of selected metabolites (**Figures 6D–F**) were consistent with the metabolome data (**Figures 6A–C**), which proved the reliability of metabolome data.

### 3.4 Conjoint analysis of transcriptome and metabolome

**Figure 7:**
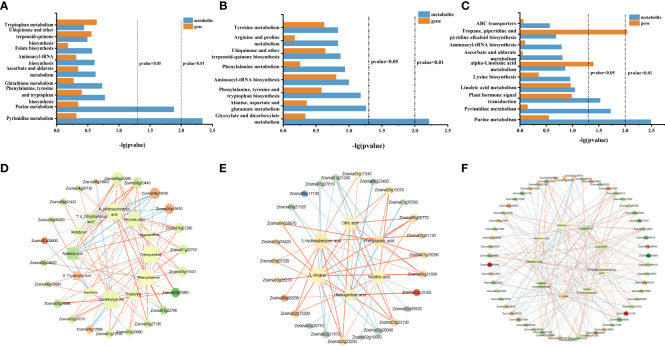
KEGG co-enrichment analysis based on the transcriptome and metabolome data in roots **(A)**, stems **(B)**, and leaves **(C)**. Results of the correlation analysis between DEGs and metabolites in roots **(D)**, stems **(E)**, and leaves **(F)**. Positive correlations are indicated by an orange line, and negative correlations are indicated by a blue line. KEGG, Kyoto Encyclopedia of Genes and Genomes; DEGs, differentially expressed genes.

In order to study the common enriched pathways of DEGs and DAMs, we performed a KEGG co-enrichment analysis. The results showed nine co-enrichment pathways in roots, including pyrimidine metabolism, purine metabolism, phenylalanine tyrosine, tryptophan biosynthesis, and other metabolic pathways (**Figure 7A**). There were eight co-enrichment pathways in stems, including glyoxylate and dicarboxylate metabolism, ubiquinone and other terpenoid-quinone biosynthesis, arginine and proline metabolism, and other metabolic pathways (**Figure 7B**). There were 10 co-enrichment pathways in the leaves, including purine metabolism, pyrimidine metabolism, plant hormone signal transduction, tropane piperidine, pyridine alkaloid biosynthesis, and other metabolic pathways (**Figure 7C**). These results indicated that these metabolic pathways may jointly participate in the process of NO regulation in eelgrass.

To further understand the relationship between DEGs and DAMs, the correlation analysis was performed based on Spearman’s correlation coefficient. In roots, the gene involved in pyrimidine metabolism (Zosma03g12850) was negatively correlated with most metabolites, while the gene involved in purine metabolism (Zosma03g05960) was positively associated with most metabolites and genes in the jasmonic acid-related pathway, transmembrane transporter activity, and symporter activity pathway (Zosma06g25690 and Zosma01g01290) were strongly positive correlated with DAMs in the phenylalanine tyrosine and tryptophan biosynthesis and glutathione metabolism (chlorogenic acid and 4-hydroxycinnamic acid) (**Figure 7D**). In addition, several DEGs (Zosma04g18610, Zosma01g25830, and Zosma04g18620) involved in the flavonoid biosynthesis pathway and naringin–chalcone synthase activity were negatively correlated with DAMs. These results showed that when NO synthesis was blocked, the expression of genes related to jasmonic acid, flavonoid biosynthesis, and transmembrane transport decreased, which led to a decrease in the accumulation of metabolites downstream. In stems, the DEGs (Zosma01g39290, Zosma06g00770, and Zosma02g10160) involved in glycolysis, glyoxylate, and dicarboxylate metabolism were positively correlated with many DAMs, such as citric acid, isocitric acid, and l-proline. In addition, there was a strong negative correlation between the DEGs in metal ion transport and ion homeostasis-related pathways (Zosma05g21520 and Zosma01g35590) with the homogentisic acid (HGA) and proline (**Figure 7E**). These results indicated that ion transport, ion homeostasis, and energy-related pathways were significantly affected after NO synthesis was inhibited. In leaves, the DEGs involved in purine metabolism, pyrimidine metabolism, plant hormone signal transduction, and MAPK signaling pathway-plant (Zosma03g05960, Zosma03g22290, Zosma05g12850, Zosma03g35940, and Zosma01g41250) were negatively correlated with DAMs in many pathways. In addition, there was a strong positive correlation between the DEGs in response to auxin, auxin-activated signaling pathway, calmodulin binding, tropane piperidine, and pyridine alkaloid biosynthesis pathways (Zosma01g13700, Zosma05g13460, Zosma05g26860, and Zosma04g01420) with DAMs (**Figure 7F**). These results indicated that NO in leaves has a significant impact on purine and pyrimidine metabolism and plant hormone signal transduction.

## 4 Discussion

### 4.1 Conjoint analysis to explore the role of NO in adapting to high-salinity marine environment in roots of eelgrass

Jasmonic acid is an important plant hormone and signal molecule in plant defense response. When plants are subjected to abiotic stress, jasmonic acid can effectively induce the expression of defense system genes, thereby enhancing the tolerance of plants to stress. In this study, we found that the downregulated genes of roots involved in the jasmonic acid-related pathway were significantly enriched after inhibiting NO synthesis (**Figure 2A**), including WRKY transcription factor 7 (WRKY7) (Zosma05g26860), allene oxide synthase (AOS) (Zosma01g01290), 3-ketoacyl-CoA thiolase 2 (KAT2) (Zosma06g19680), and 4-coumarate coenzyme A ligase (4CL) (Zosma06g25690) (**Supplementary Table 2**). As one of the most important transcription factor families in higher plants, WRKY transcription factors can regulate the expression of downstream functional genes and then participate in the defense processes against biotic and abiotic stress, growth, and development. There are *cis*-acting elements related to hormone regulation, biotic stress, and abiotic stress in the WRKY gene family of terrestrial plants (Cheng et al., 2021). For example, CsWRKY7 in tea plants is mainly involved in growth and the response of plants to environmental stress (Chen et al., 2019). Similar to terrestrial plants, the *cis*-acting elements mentioned above also exist in the WRKY gene family in the eelgrass (unpublished data), indicating that the WRKY gene family in the eelgrass may perform similar functions to terrestrial plants. AOS and KAT2 are key enzymes in the jasmonic acid biosynthesis pathway, which regulate the defense response by influencing the biosynthesis of jasmonic acid (Castillo et al., 2004; Sun et al., 2023). 4CL is involved in synthesizing different phenylpropanoid derivatives (Zhang et al., 2019). 4CL is the main enzyme at the branch point, which plays an important role in the synthesis and accumulation of flavonoids by converting the phenylpropanoid biosynthesis pathway into the flavonoid biosynthesis pathway. Flavonoids exhibit strong antioxidant defense biological activity and are used by organisms to cope with almost all abiotic stresses (Zhang et al., 2019). Through the analysis of the correlation network diagram, we found a strong positive correlation between WRKY7 (Zosma05g26860) and AOS (Zosma01g01290) (**Supplementary Figure 5A**). Therefore, we speculated that the NO synthesis pathway might participate in transcriptional regulation through the transcription factor WRKY7, which activates the downstream AOS gene to synthesize jasmonic acid and participate in the adaptation to high-salt environments. The 4CL in the jasmonic acid biosynthesis pathway affects the synthesis of flavonoids by regulating phenylpropane biosynthesis. Interestingly, we also discovered that the expression levels of genes involved in phenylpropanoid biosynthesis, flavonoid biosynthesis, flavone and flavonol biosynthesis, and naringenin-chalcone synthase activity pathways in eelgrass (**Figure 2B**), such as *p*-coumaroyl shikimate 3-hydroxylase (C3′H), 4CL, and flavonoid glucosyltransferase family GT1, were significantly downregulated. These genes are related to the synthesis of phenylpropanoids and flavonoids in terrestrial plants (Coleman et al., 2008; Zhang et al., 2019; Zhang et al., 2020; Ma et al., 2022). Through the analysis of the correlation network diagram, we found that the genes mentioned above, which are involved in phenylpropanoid biosynthesis pathway, flavonoid biosynthesis pathway (Zosma02g24370, Zosma04g20730, and Zosma06g25690), and jasmonic acid biosynthesis pathway (Zosma05g26860, Zosma01g01290, and Zosma06g1968) had a strong positive correlation (**Supplementary Figure 5A**). There was a strong positive correlation between l-phenylalanine, 4-hydroxycinnamic acid (4-HCA), and genes in the phenylpropanoid and jasmonic acid biosynthesis pathways (Zosma06g25690 and Zosma01g01290) (**Figure 7D**). At the same time, the contents of l-phenylalanine, 4-HCA, and chlorogenic acid in the phenylalanine synthesis pathway of roots were downregulated (**Supplementary Table 5**). This further proved that our speculation was reasonable. In the phenylpropanoid biosynthesis pathway, phenylalanine was used as the initial reaction substrate and then intermediate metabolites (such as cinnamic acid, coumaric acid, and chlorogenic acid) generated after various reactions and transformed into secondary metabolites (such as lignin, flavonoids, and alkaloids) (Vogt, 2010; Fraser and Chapple, 2011; Pratyusha and Sarada, 2022). Chlorogenic acid and 4-HCA have the effects of antioxidants, scavenging free radicals, and preventing apoptosis induced by oxidative stress, which can improve the ability of terrestrial plants to adapt to adversity and maintain their normal growth and development (Liang and Kitts, 2015; Yan et al., 2016; Yu and Fan, 2021). In addition, the contents of metabolites, such as melatonin, ascorbic acid (ASA), and allantoic acid, were also reduced (**Supplementary Table 5**). These metabolites with antioxidant functions have been shown to be able to cope with abiotic oxidative damage and play an important role in maintaining the integrity of cell membranes by removing reactive oxygen and free radicals in terrestrial plants (Brychkova et al., 2008; Gallie, 2013; Li J. et al., 2019). Therefore, NO in the eelgrass roots could regulate the expression of genes in jasmonic acid biosynthesis and phenylpropanoid biosynthesis to regulate the contents of metabolites with antioxidant and free radical scavenging functions to enhance the antioxidant defense ability and improve the adaptation of eelgrass to high-salt environments.

The results showed that DEGs in various functional pathways related to transmembrane transporter activity were significantly enriched (**Figure 2A**), and the contents of l-phenylalanine and cysteinylglycine were also significantly downregulated. Moreover, there was a strong positive correlation between these DEGs and DAMs (**Figure 7B**), indicating that these amino acids may be regulated by genes related to transmembrane transport activity. When plants are subjected to abiotic stress, these amino acids act as osmotic regulators to regulate osmotic pressure and maintain cell membrane stability to prevent water loss in terrestrial plants (Batista-Silva et al., 2019; Khan et al., 2020). In addition, the content of betaine, an osmotic adjustment substance, was also downregulated. These results indicated that the NO synthesis could regulate the osmotic balance in roots and maintain the stability of the cell membrane, thus enhancing the adaptation to high-salt environments.

**Figure 8:**
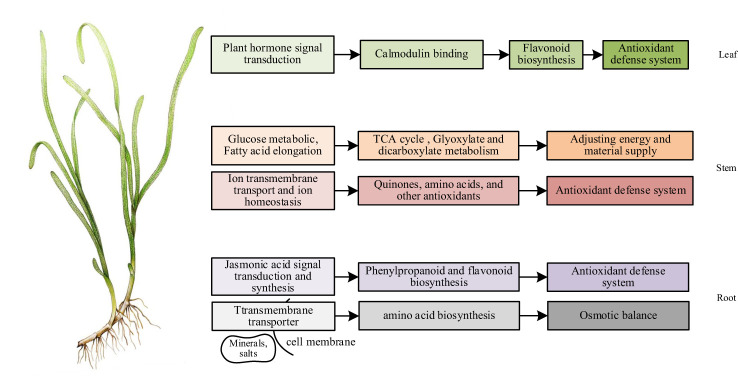
The mechanism of NO affecting the adaptability of eelgrass to high-salt environment (Website China Dialogue Ocean, 2020).

The conjoint analysis of transcriptome and metabolome in roots showed that the effect of NO on eelgrass was similar to that in terrestrial plants. NO mainly regulated the downstream phenylpropanoid metabolic pathway through the pathway related to the biosynthesis of jasmonic acid, entered the flavonoid branch, and finally improved the adaptability of eelgrass roots to high-salt environments through the antioxidant defense system. In addition, genes involved in transmembrane transport pathways regulated the synthesis of several amino acids, thereby maintaining the osmotic pressure balance and enhancing the adaptability of eelgrass to high-salt environments (**Figure 8**).

### 4.2 Conjoint analysis to explore the role of NO in adapting to high-salinity marine environment in stems of eelgrass

Maintaining intracellular ion homeostasis is an essential feature for plants to adapt to high-salt environments. When plants are exposed to high-salt environments, excessive accumulation of harmful ions (such as Na^+^) in cells will affect normal metabolic activities, such as the absorption of other ions (such as K^+^), resulting in an imbalance of ion homeostasis (Yin et al., 2023). In this study, the expression levels of genes such as ABC transporter B family member 21 (Zosma06g17130), zinc transporter (ZTP) (Zosma01g21230), zinc transporter ZTP29 (Zosma05g21520), and manganese transport protein MntH (Zosma01g35590) (**Supplementary Table 3**) in ABC transporter, zinc ion transmembrane transporter, and iron ion transporter pathways in the stems were significantly downregulated after inhibiting NO synthesis (**Figures 2C, D**). Studies on terrestrial plants have shown that ABC transporters can transport various substances, among which subfamily B can transport heavy metal ions, which can avoid cell toxicity to heavy metal ions (Kim et al., 2006; Chen et al., 2007). ZTP can alleviate the peroxidation damage of biofilm, and the ZTP29 mutant in *Arabidopsis thaliana* is involved in the response to salt stress by regulating Zn content (Wang et al., 2010). The manganese transporter affects the absorption and transport of Mn, as well as plays a role in oxidative stress (Ha and Lee, 2023). Theoretically, similar to terrestrial plants, NO regulates the ion balance in stems by affecting various transporters to participate in the antioxidant defense system and ultimately improve the adaptability of eelgrass to high-salt environments. Interestingly, we found that the contents of metabolites that can clear reactive oxygen, such as HGA (Rosa et al., 2011), curcumol (Peng et al., 2021), mulberrin (Ge et al., 2022), and 6-hydroxydaidzein (Esaki et al., 1999), were significantly upregulated (**Supplementary Table 7**). These antioxidant metabolites are currently primarily used in pharmacological studies but have not been found to play a role in plant adaptation to abiotic stress, indicating that they may be unique metabolites in eelgrass with the function of adapting to high-salt environments. We discovered that genes involved in ion homeostasis and transmembrane transport pathways (Zosma05g21520 and Zosma01g35590) were strongly negatively correlated with HGA (**Figure 7E**). This indicated that NO may affect antioxidant metabolites such as HGA through transmembrane transport, enhancing adaptability to the high-salt environment in eelgrass stems.

Carbohydrates such as glucose are the foundation of plant growth and development. This study revealed that the NO synthesis pathway in eelgrass also affected genes involved in the glucose metabolic process and glyceraldehyde-3-phosphate dehydrogenase (NAD^+^) activity (**Supplementary Figure 2C**), and the significantly enriched glyceraldehyde-3-phosphate dehydrogenase (phosphorylating) (GAPDH) gene was upregulated (**Supplementary Table 3**). GAPDH is a glycolytic enzyme and also an inhibitor of glycolytic activity (Qvit et al., 2016). Glycolysis is the primary metabolic pathway of glucose oxidation, providing energy and intermediate precursors for other metabolic pathways. In addition, the experimental results showed that the fatty acid elongation pathway was also affected (**Figure 2D**). Fatty acids generate acetyl-CoA through various mitochondrial decomposition β-oxidation processes and then enter the TCA cycle to produce ATP. It was worth noting that the TCA cycle, glyoxylate, and dicarboxylate metabolism in the stems of eelgrass were also significantly affected (**Figure 5C**), and the contents of enriched metabolites, citric acid, and isocitric acid, were significantly upregulated (**Supplementary Table 7**). Glyoxylate and dicarboxylate metabolism, known as the supply pathway of the TCA cycle, provides energy for plants (Schnarrenberger and Martin, 2002). The TCA cycle is a hub connecting the three major metabolic pathways of carbohydrates, lipids, and amino acids, promoting amino acid metabolism and energy cycling under salt stress (Widodo et al., 2009). Previous studies on terrestrial plants have revealed that the TCA cycle pathway was promoted, and intermediate metabolites (malic acid and citric acid) were significantly accumulated under salt stress conditions (Panda et al., 2021). Citric acid is an antioxidant, and its high accumulation contributes to improving the tolerance and adaptability of plants to abiotic stress (Tahjib-Ul-Arif et al., 2021). These results suggested that after the NO synthesis was inhibited, eelgrass enhanced its adaptability to the high-salt environment by accumulating citric acid to promote the TCA cycle. Interestingly, there was a strong positive correlation between the GAPDH and isocitric acid through correlation network analysis (**Supplementary Figure 5B**). This indicated that the glyoxylate and dicarboxylate metabolism and the TCA cycle would be regulated as complementary possibly while the glycolytic process had been inhibited in the stems of eelgrass, which can provide sufficient energy and raw materials for metabolite synthesis to adapt to high-salt environments.

In summary, the results of conjoint analysis showed that the function of NO in the stems of eelgrass was similar and unique to that of terrestrial plants. The similarity was that NO in the stems also plays a vital role in salt tolerance through metal ion transport and ion homeostasis. The difference was that NO in the stems of eelgrass may regulate unique substances with antioxidant function *in vivo* to enhance the antioxidant defense system so that eelgrass can continue to adapt to high-salt environments. In addition, after the NO synthesis pathway was inhibited, glucose metabolism helped the stems of eelgrass continue to adapt to high-salt environments by regulating glyoxylic acid and dicarboxylic acid metabolism and TCA cycling as complementary effects (**Figure 8**).

### 4.3 Conjoint analysis to explore the role of NO in adapting to high-salinity marine environment in leaves of eelgrass

Plant hormone signaling pathways play a crucial regulatory role in plant response to various stresses (Waadt et al., 2022). Auxin is a plant hormone that plays a key role in many aspects of plant development and is an important signal for response to stress (Jing et al., 2023). In this study, the results showed that the expression levels of genes in response to auxin, auxin-activated signaling pathway, plant hormone signal transduction pathway, and MAPK signaling pathway-plant (**Figure 2E**), such as ABC transporter B family member 19 (ABCB19) (Zosma06g04050), auxin signaling F-box 2 (AFB2) (Zosma01g13700), and auxin-responsive protein (Zosma01g08410 and Zosma05g13460) were significantly downregulated after the NO synthesis pathway was inhibited (**Supplementary Table 4**). In terrestrial plants, ABCB19 can efficiently transport auxin (Ma and Han, 2016), AFB2 plays an essential role in auxin polar transport (Guo et al., 2021), and the auxin-responsive protein family plays a central role in auxin signal transduction by interacting with auxin-responsive factors (Li et al., 2020). In addition, this study revealed that the eelgrass calmodulin binding pathway was also affected by NO, such as the expression levels of calmodulin-binding protein (Zosma03g19950), IQ-domain 6 (IQ6) (Zosma06g11260), and putative cyclic nucleotide-gated ion channel 9 (CNGC9) (Zosma06g17940), which were significantly downregulated (**Supplementary Table 4**), suggesting that NO plays a role in calcium signal transduction. As the second messenger of plants, Ca^2+^ is widely involved in stress response and developmental regulation processes (Tong et al., 2021). Previous studies showed that Ca^2+^ in plants is closely related to NO. NO can regulate the concentration of Ca^2+^, while the synthesis of NO depends on Ca^2+^ (Besson-Bard et al., 2008; Wu et al., 2021). When NO synthesis was inhibited in the presence of Ca^2+^, the growth traits of *Brassica* seedlings were significantly inhibited (Singh et al., 2020). NO treatment increased the expression of calmodulin (CAM) and calcium protein kinase (CPK) genes in *Brassica napus* under salt stress (Rezayian and Zarinkamar, 2023). However, Ca^2+^ also affects the synthesis of NO, acting as a promoter and a sensor in NO signaling pathways (Mariyam et al., 2023). Interestingly, the gene correlation network analysis showed that genes in the auxin-activated signaling pathway (Zosma01g08410, Zosma05g13460, Zosma01g26740, and Zosma01g42050) and calmodulin binding pathway (Zosma03g19950, Zosma04g01420, Zosma05g26860, and Zosma06g11260) had a strong positive correlation (**Supplementary Figure 5C**). Research on terrestrial plants has shown there is a close relationship between Ca^2+^ and auxin. Auxin polar transport is highly dependent on calcium signal transduction, and auxin is an effective inducer of Ca^2+^ signaling (Li T. et al., 2019; Winnicki, 2020). These results indicated that the plant hormones and Ca^2+^ signaling pathways in the eelgrass leaves were significantly affected after the NO synthesis of eelgrass was inhibited, which is similar to terrestrial plants, and there was a certain regulatory relationship between them. The specific mechanism needs further research in the future. Surprisingly, the gene correlation network analysis showed that genes in the auxin-related pathway and the calmodulin-binding protein pathway (Zosma01g26740, Zosma05g26860, Zosma06g11260, and Zosma06g17940) and flavonoid biosynthesis (Zosma06g08180 and Zosma06g25630) had a strong positive correlation (**Supplementary Figure 5C**). Studies in *Arabidopsis* have shown that auxin response factor 2 plays a positive regulatory role in the synthesis and accumulation of flavonoids (Jiang et al., 2022). This revealed that similar to terrestrial plants, NO also regulates antioxidant substances such as flavonoids in response to high-salt environments in eelgrass leaves.

In short, we speculated that NO can affect plant hormone signaling pathways and calcium-binding protein pathways in the leaves through the combined transcriptome and metabolome analysis, thereby affecting downstream gene expression and metabolite synthesis to enhance the adaptability to the high-salt environment through antioxidant regulation in eelgrass (**Figure 8**).

For terrestrial plants, the roots absorb water and inorganic salts from the soil and transport them to the stems and leaves. For seagrass living in the ocean, the leaf is the primary tissue that absorbs inorganic salts. The physiological functions of different tissues of land plants and seagrass are significantly different, and the role of NO in different tissues is also different. It was worth noting that we discovered some unique DEGs and DAMs in this study. Transcriptome data showed that TBC1 domain family member 8B (Zosma01g17090) was found in the DEG analysis of all tissues in eelgrass. This gene has been associated with cancer research in clinicopathology (Liu et al., 2022) and is mainly involved in protein coding, and its function in plants has not yet been discovered. Cleft lip and palate transmembrane protein (Zosma01g41810) and adiponectin receptor protein (Zosma01g32560) were also found in roots and stems of eelgrass, respectively, which were also mostly related to pathological studies (Kim et al., 2018; Zhang et al., 2023). Protein ApaG (Zosma01g01070) with unknown function was found in the leaves of eelgrass. Metabolome analysis showed that there were several unique antioxidant functional substances in stems, such as HGA, curcumol, mulberrin, and 6-hydroxydaidzein. The above genes and metabolites may play a unique regulatory role in the adaptation of eelgrass to marine high-salt environments, and the specific mechanism needs to be further studied in the future. This article studies the salt tolerance mechanism of eelgrass through omics technology, providing practical reference for the study of molecular adaptation mechanisms of marine plants and providing theoretical support for salt tolerance research, reproduction of other marine plants, and improvement of salt tolerance of terrestrial crops in the future, for example, verification of the role of DEGs in other plants through cloning technology and heterologous expression experiments and verification of the specific role of DAMs in other plants through physiological and biochemical experiments in the future.

## 5 Conclusions

In this study, we sequenced and analyzed the transcriptome and metabolome in eelgrass roots, stems, and leaves. A total of 326, 368, and 859 DEGs and 63, 52, and 36 DAMs were identified from roots, stems, and leaves, respectively. The combined transcriptome and metabolome data provided insight into the association between expression regulation of important genes and metabolites. In this study, the conjoint analysis uncovered the regulatory pathway of NO in different tissues of eelgrass. In the roots and stems, NO enhanced the adaptability of eelgrass to high-salt environments through antioxidant defense ability. The difference is that NO in the roots and stems can also regulate osmotic balance and energy metabolism, respectively, to improve the adaptability of eelgrass to high-salt environments. The leaves ultimately affected the synthesis of antioxidants through plant hormone signal transduction to cope with high-salt environments. Seagrass has a close genetic relationship with crops. In the future, we can further carry out gene cloning and physiological function research, verify the functions of genes in key pathways, and use transgenic technology to improve crop salt resistance traits.

## Data availability statement

The datasets presented in this study can be found in online repositories. The names of the repository/repositories and accession number(s) can be found below: NCBI Accession number: SRR26157017 - SRR26157034.

## Author contributions

XW: Investigation, Project administration, Visualization, Validation, Writing – original draft, Writing – review & editing. TW: Data curation, Project administration, Validation, Writing – review & editing. PY: Data curation, Writing – review & editing. YL: Project administration, Supervision, Writing – review & editing. XL: Conceptualization, Methodology, Project administration, Supervision, Writing – review & editing.
