# Supplementary material for: NO enhances the adaptability to high-salt environments by regulating osmotic balance, antioxidant defense, and ion homeostasis in eelgrass based on transcriptome and metabolome analysis
Source: Front Plant Sci. 2024 Feb 7;15:1343154. doi: 10.3389/fpls.2024.1343154 (PMC10880190; doi:10.3389/fpls.2024.1343154)
Supplement: Supplementary file 1 [file Presentation_1.pptx]

## Slide 1
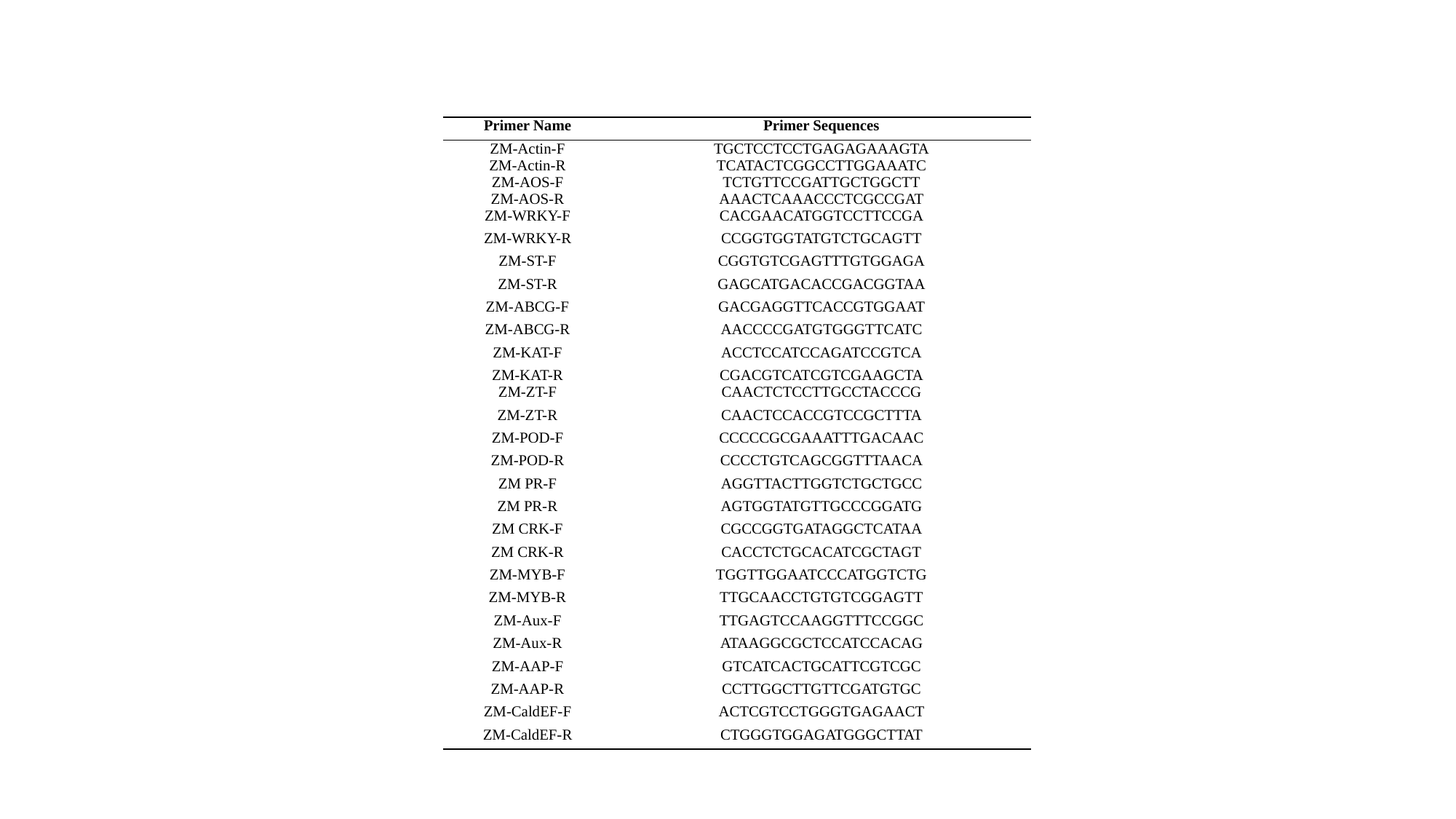

| Primer Name | Primer Sequences |
| --- | --- |
| ZM-Actin-F | TGCTCCTCCTGAGAGAAAGTA |
| ZM-Actin-R | TCATACTCGGCCTTGGAAATC |
| ZM-AOS-F | TCTGTTCCGATTGCTGGCTT |
| ZM-AOS-R | AAACTCAAACCCTCGCCGAT |
| ZM-WRKY-F | CACGAACATGGTCCTTCCGA |
| ZM-WRKY-R | CCGGTGGTATGTCTGCAGTT |
| ZM-ST-F | CGGTGTCGAGTTTGTGGAGA |
| ZM-ST-R | GAGCATGACACCGACGGTAA |
| ZM-ABCG-F | GACGAGGTTCACCGTGGAAT |
| ZM-ABCG-R | AACCCCGATGTGGGTTCATC |
| ZM-KAT-F | ACCTCCATCCAGATCCGTCA |
| ZM-KAT-R | CGACGTCATCGTCGAAGCTA |
| ZM-ZT-F | CAACTCTCCTTGCCTACCCG |
| ZM-ZT-R | CAACTCCACCGTCCGCTTTA |
| ZM-POD-F | CCCCCGCGAAATTTGACAAC |
| ZM-POD-R | CCCCTGTCAGCGGTTTAACA |
| ZM PR-F | AGGTTACTTGGTCTGCTGCC |
| ZM PR-R | AGTGGTATGTTGCCCGGATG |
| ZM CRK-F | CGCCGGTGATAGGCTCATAA |
| ZM CRK-R | CACCTCTGCACATCGCTAGT |
| ZM-MYB-F | TGGTTGGAATCCCATGGTCTG |
| ZM-MYB-R | TTGCAACCTGTGTCGGAGTT |
| ZM-Aux-F | TTGAGTCCAAGGTTTCCGGC |
| ZM-Aux-R | ATAAGGCGCTCCATCCACAG |
| ZM-AAP-F | GTCATCACTGCATTCGTCGC |
| ZM-AAP-R | CCTTGGCTTGTTCGATGTGC |
| ZM-CaldEF-F | ACTCGTCCTGGGTGAGAACT |
| ZM-CaldEF-R | CTGGGTGGAGATGGGCTTAT |

## Slide 2
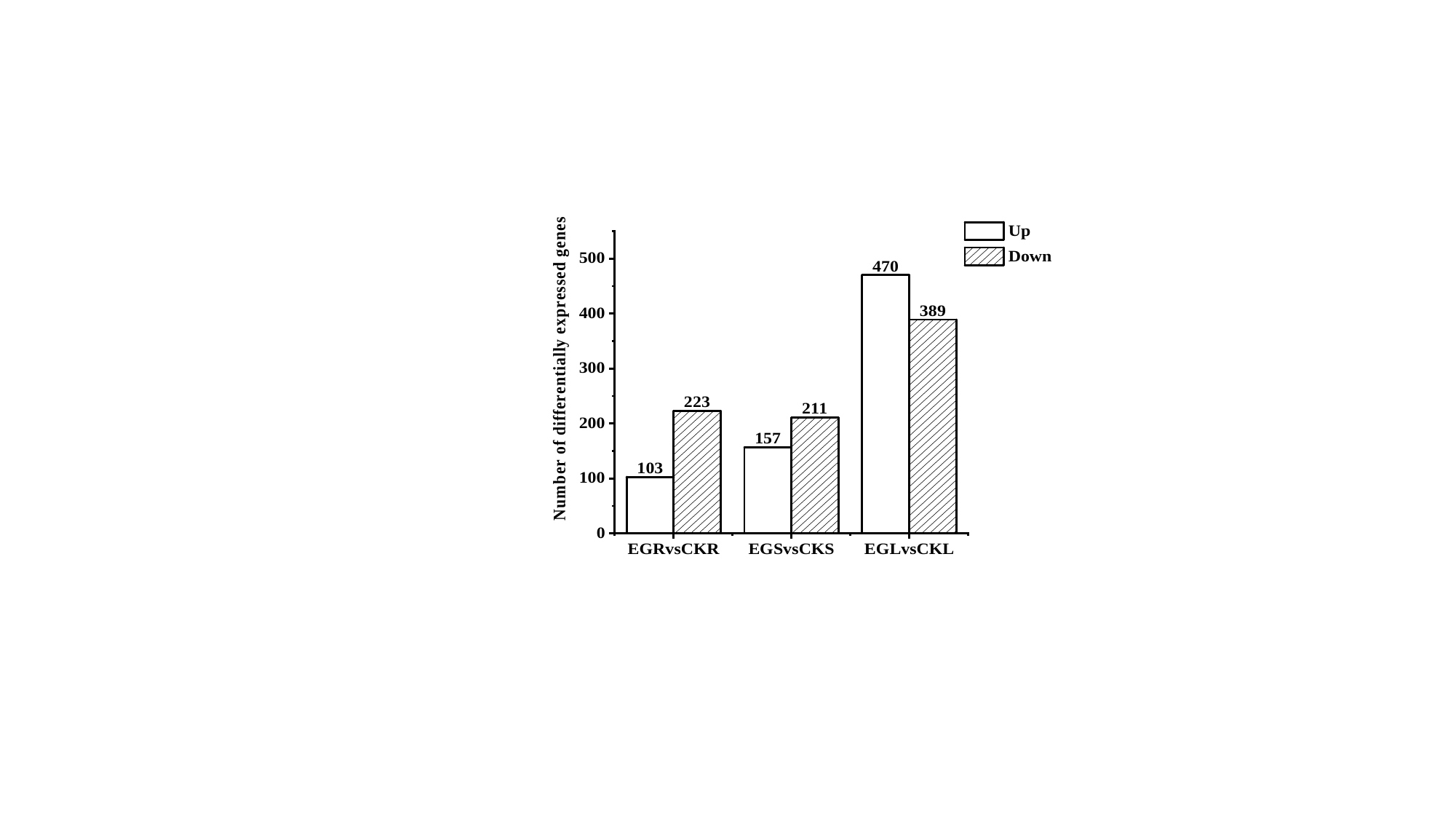

## Slide 3
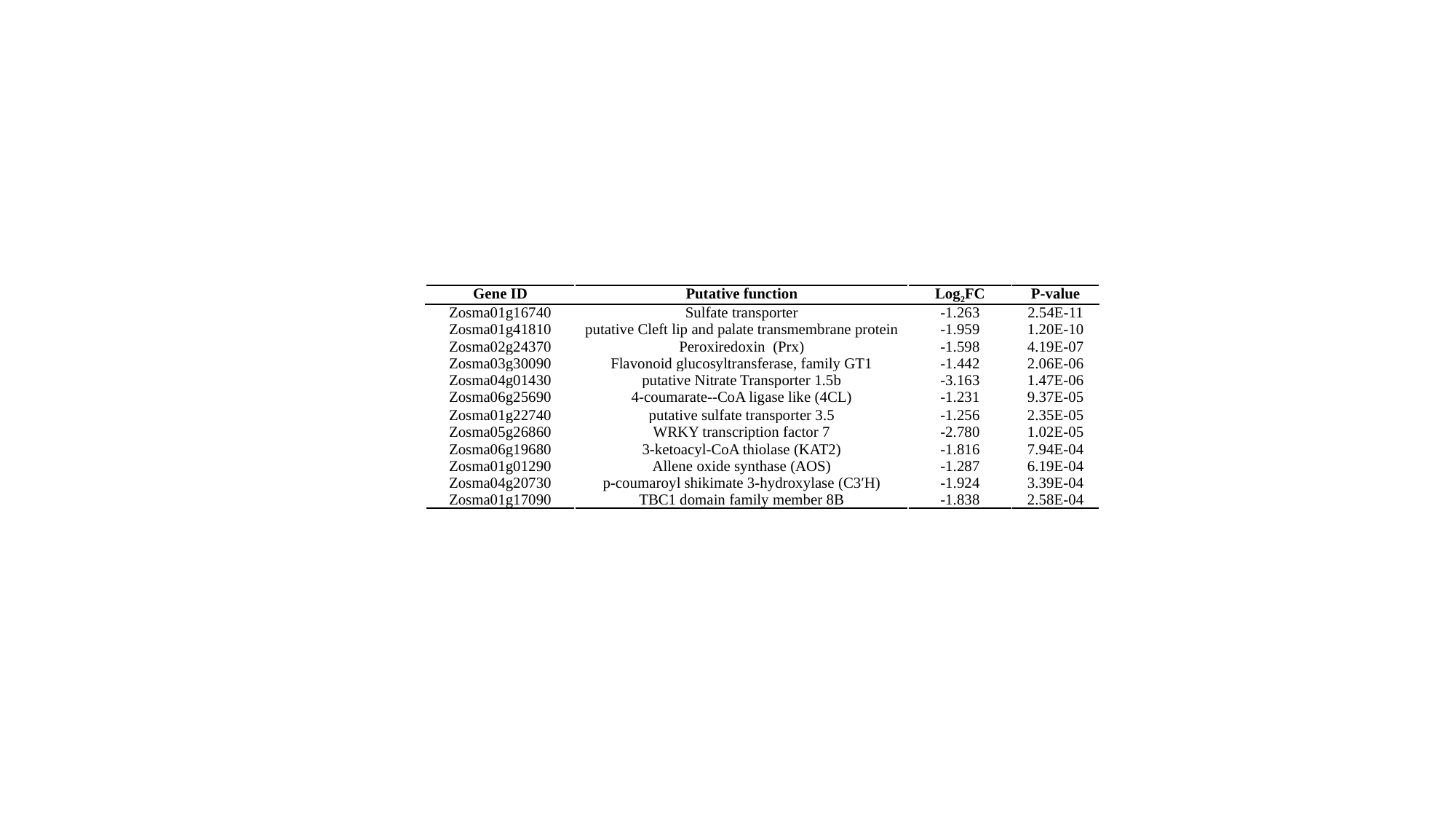

| Gene ID | Putative function | Log2FC | P-value |
| --- | --- | --- | --- |
| Zosma01g16740 | Sulfate transporter | -1.263 | 2.54E-11 |
| Zosma01g41810 | putative Cleft lip and palate transmembrane protein | -1.959 | 1.20E-10 |
| Zosma02g24370 | Peroxiredoxin (Prx) | -1.598 | 4.19E-07 |
| Zosma03g30090 | Flavonoid glucosyltransferase, family GT1 | -1.442 | 2.06E-06 |
| Zosma04g01430 | putative Nitrate Transporter 1.5b | -3.163 | 1.47E-06 |
| Zosma06g25690 | 4-coumarate--CoA ligase like (4CL) | -1.231 | 9.37E-05 |
| Zosma01g22740 | putative sulfate transporter 3.5 | -1.256 | 2.35E-05 |
| Zosma05g26860 | WRKY transcription factor 7 | -2.780 | 1.02E-05 |
| Zosma06g19680 | 3-ketoacyl-CoA thiolase (KAT2) | -1.816 | 7.94E-04 |
| Zosma01g01290 | Allene oxide synthase (AOS) | -1.287 | 6.19E-04 |
| Zosma04g20730 | p-coumaroyl shikimate 3-hydroxylase (C3′H) | -1.924 | 3.39E-04 |
| Zosma01g17090 | TBC1 domain family member 8B | -1.838 | 2.58E-04 |

## Slide 4
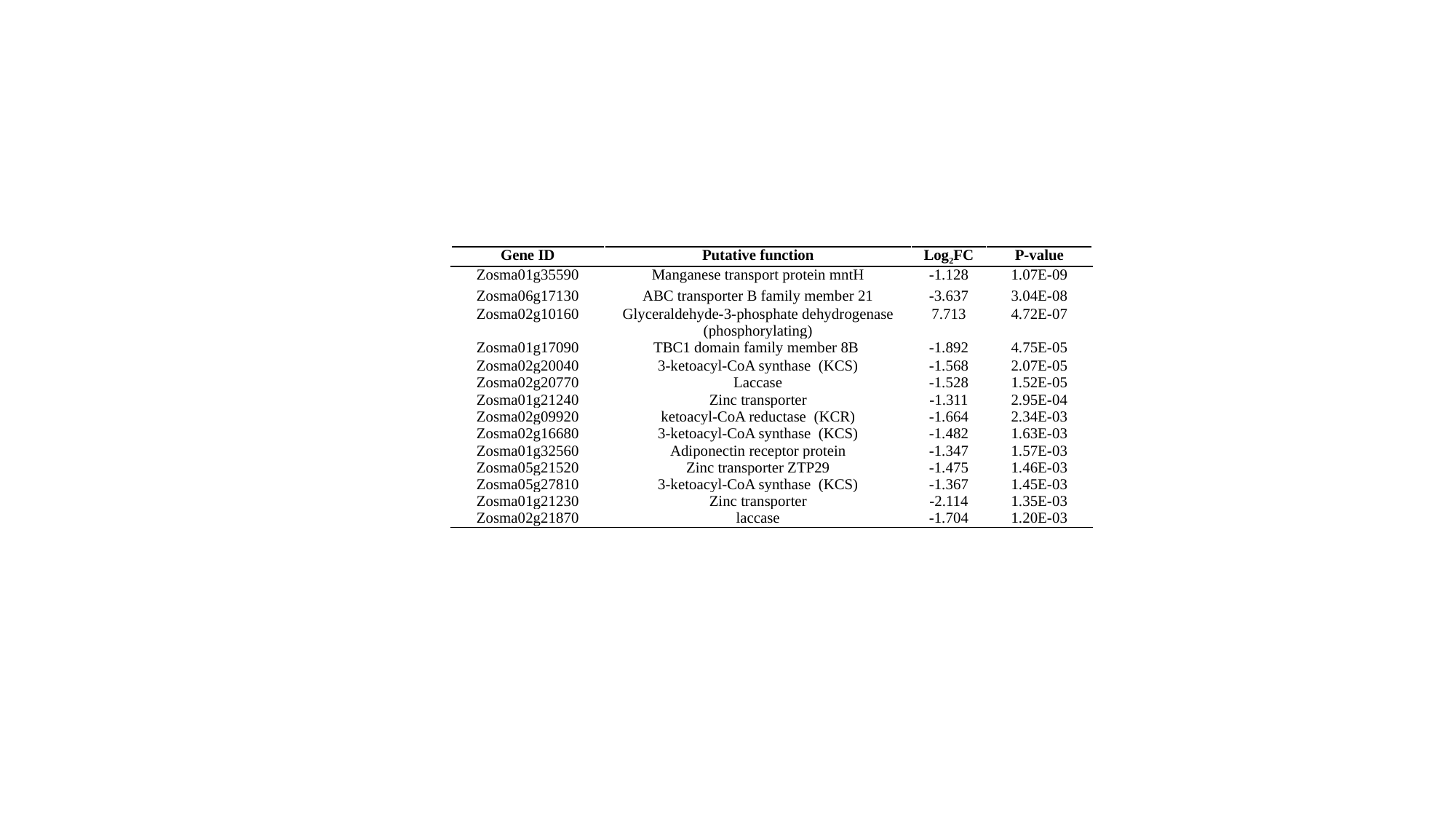

| Gene ID | Putative function | Log2FC | P-value |
| --- | --- | --- | --- |
| Zosma01g35590 | Manganese transport protein mntH | -1.128 | 1.07E-09 |
| Zosma06g17130 | ABC transporter B family member 21 | -3.637 | 3.04E-08 |
| Zosma02g10160 | Glyceraldehyde-3-phosphate dehydrogenase (phosphorylating) | 7.713 | 4.72E-07 |
| Zosma01g17090 | TBC1 domain family member 8B | -1.892 | 4.75E-05 |
| Zosma02g20040 | 3-ketoacyl-CoA synthase (KCS) | -1.568 | 2.07E-05 |
| Zosma02g20770 | Laccase | -1.528 | 1.52E-05 |
| Zosma01g21240 | Zinc transporter | -1.311 | 2.95E-04 |
| Zosma02g09920 | ketoacyl-CoA reductase (KCR) | -1.664 | 2.34E-03 |
| Zosma02g16680 | 3-ketoacyl-CoA synthase (KCS) | -1.482 | 1.63E-03 |
| Zosma01g32560 | Adiponectin receptor protein | -1.347 | 1.57E-03 |
| Zosma05g21520 | Zinc transporter ZTP29 | -1.475 | 1.46E-03 |
| Zosma05g27810 | 3-ketoacyl-CoA synthase (KCS) | -1.367 | 1.45E-03 |
| Zosma01g21230 | Zinc transporter | -2.114 | 1.35E-03 |
| Zosma02g21870 | laccase | -1.704 | 1.20E-03 |

## Slide 5
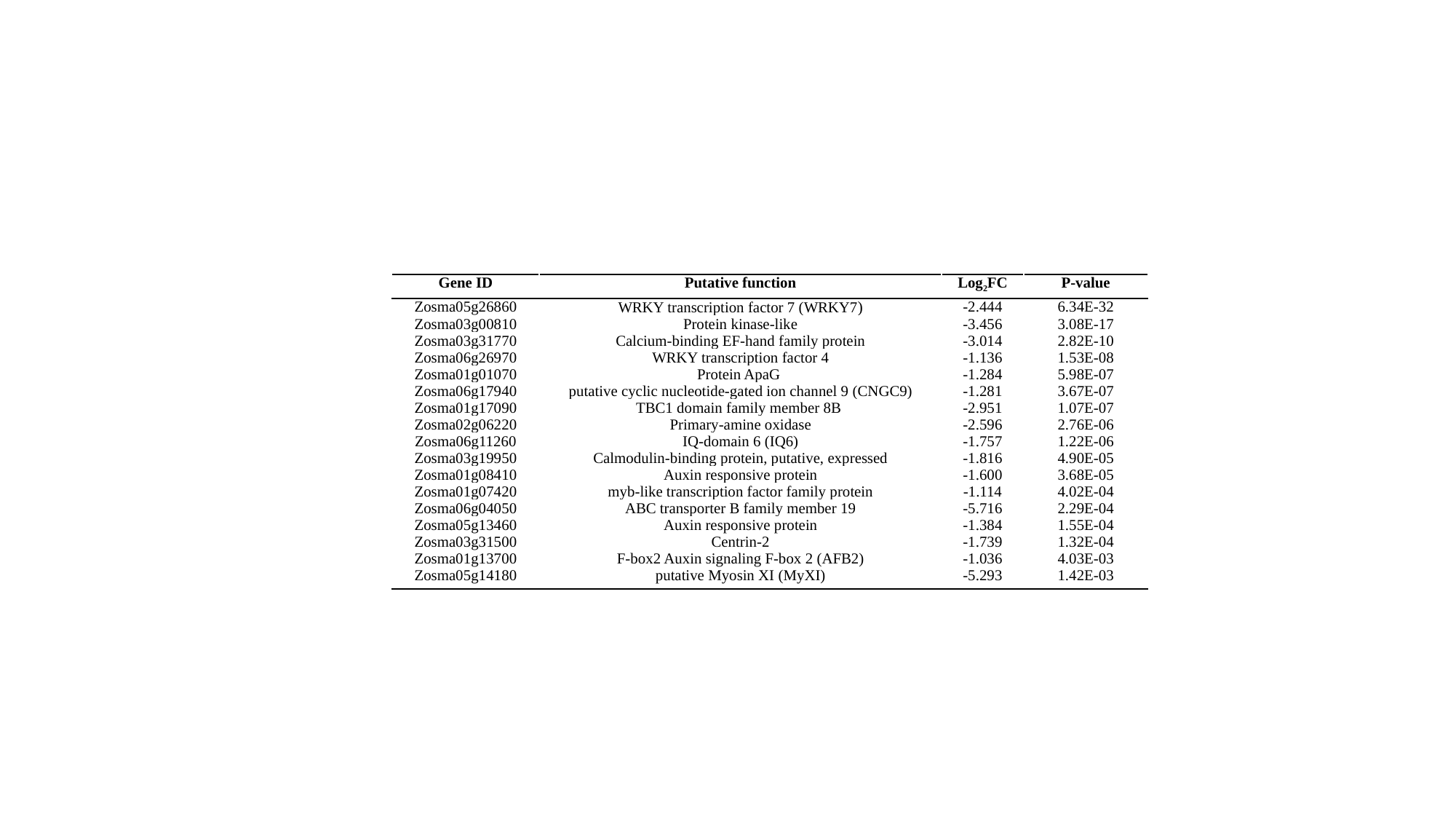

| Gene ID | Putative function | Log2FC | P-value |
| --- | --- | --- | --- |
| Zosma05g26860 | WRKY transcription factor 7 (WRKY7) | -2.444 | 6.34E-32 |
| Zosma03g00810 | Protein kinase-like | -3.456 | 3.08E-17 |
| Zosma03g31770 | Calcium-binding EF-hand family protein | -3.014 | 2.82E-10 |
| Zosma06g26970 | WRKY transcription factor 4 | -1.136 | 1.53E-08 |
| Zosma01g01070 | Protein ApaG | -1.284 | 5.98E-07 |
| Zosma06g17940 | putative cyclic nucleotide-gated ion channel 9 (CNGC9) | -1.281 | 3.67E-07 |
| Zosma01g17090 | TBC1 domain family member 8B | -2.951 | 1.07E-07 |
| Zosma02g06220 | Primary-amine oxidase | -2.596 | 2.76E-06 |
| Zosma06g11260 | IQ-domain 6 (IQ6) | -1.757 | 1.22E-06 |
| Zosma03g19950 | Calmodulin-binding protein, putative, expressed | -1.816 | 4.90E-05 |
| Zosma01g08410 | Auxin responsive protein | -1.600 | 3.68E-05 |
| Zosma01g07420 | myb-like transcription factor family protein | -1.114 | 4.02E-04 |
| Zosma06g04050 | ABC transporter B family member 19 | -5.716 | 2.29E-04 |
| Zosma05g13460 | Auxin responsive protein | -1.384 | 1.55E-04 |
| Zosma03g31500 | Centrin-2 | -1.739 | 1.32E-04 |
| Zosma01g13700 | F-box2 Auxin signaling F-box 2 (AFB2) | -1.036 | 4.03E-03 |
| Zosma05g14180 | putative Myosin XI (MyXI) | -5.293 | 1.42E-03 |

## Slide 6
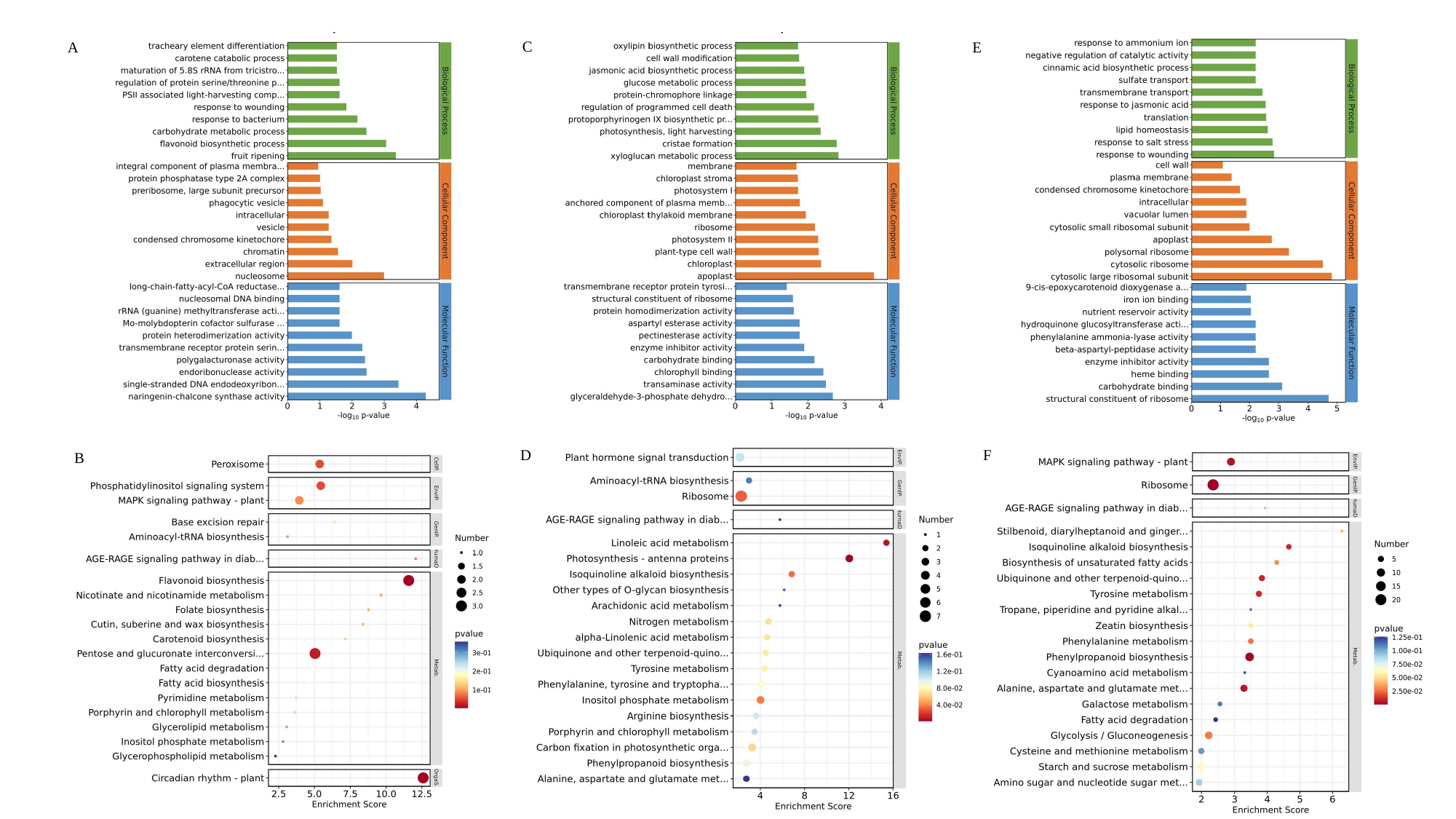

C
E
A
D
F
B

## Slide 7
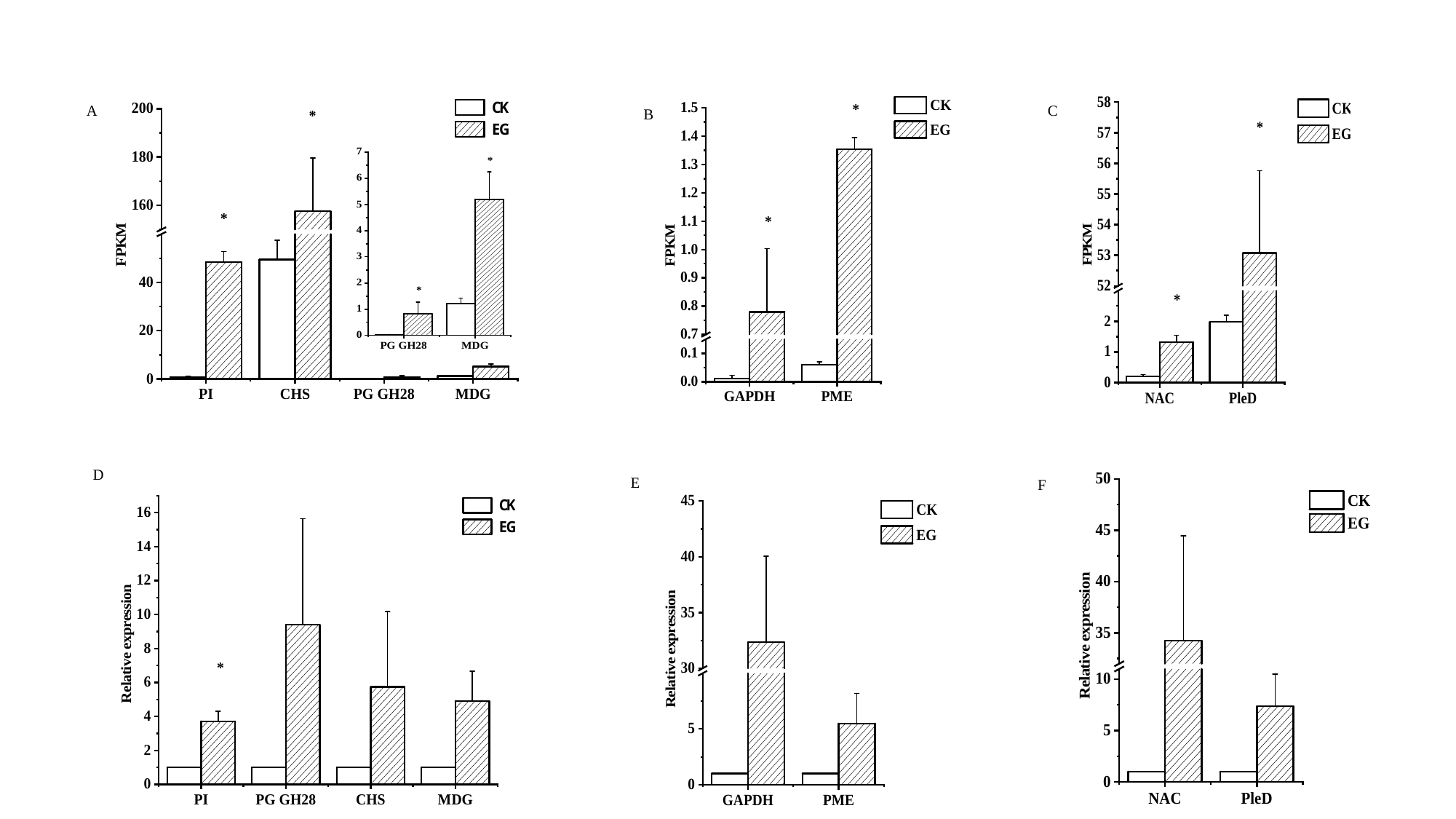

A
C
B
D
E
F

## Slide 8
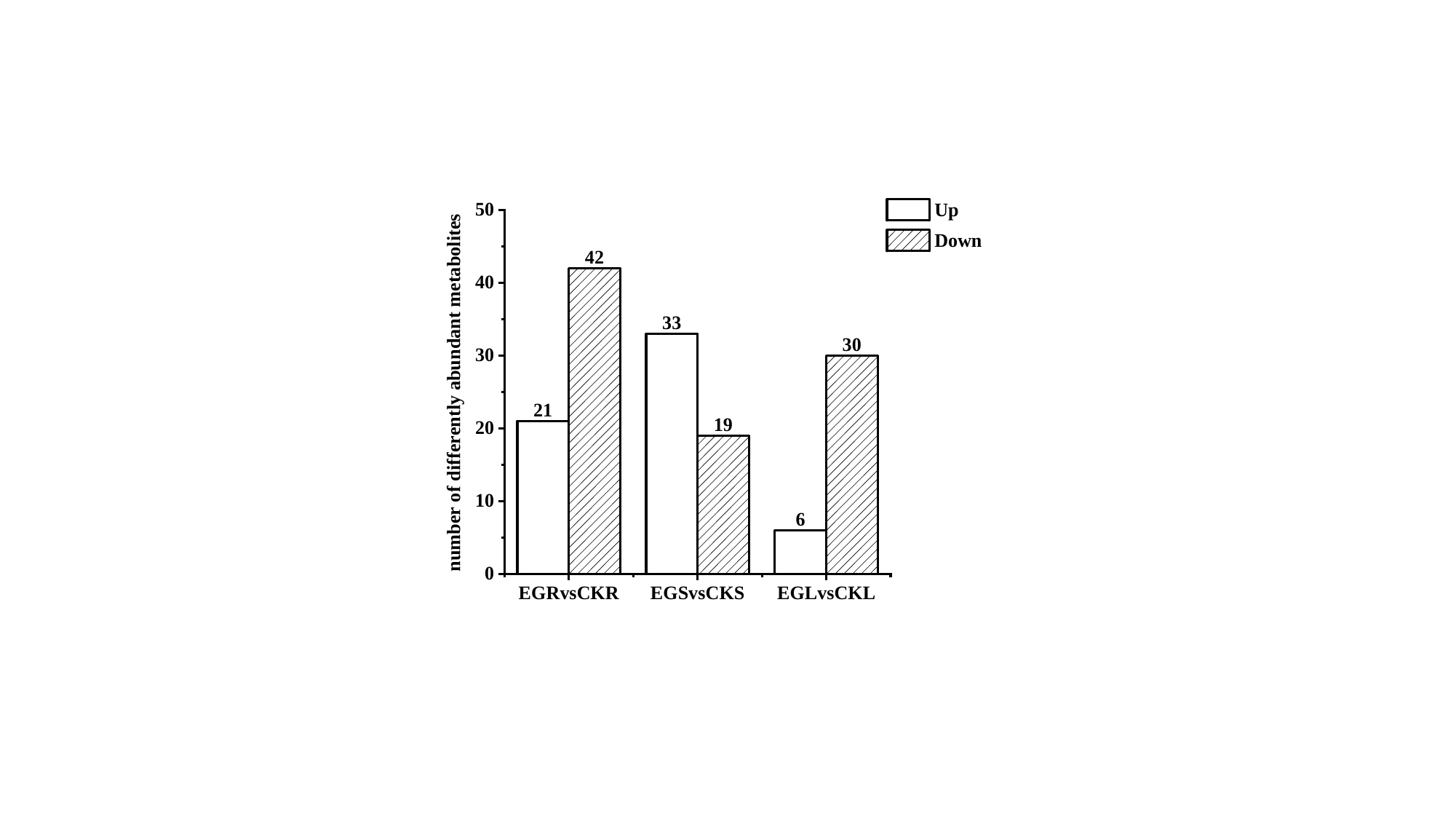

## Slide 9
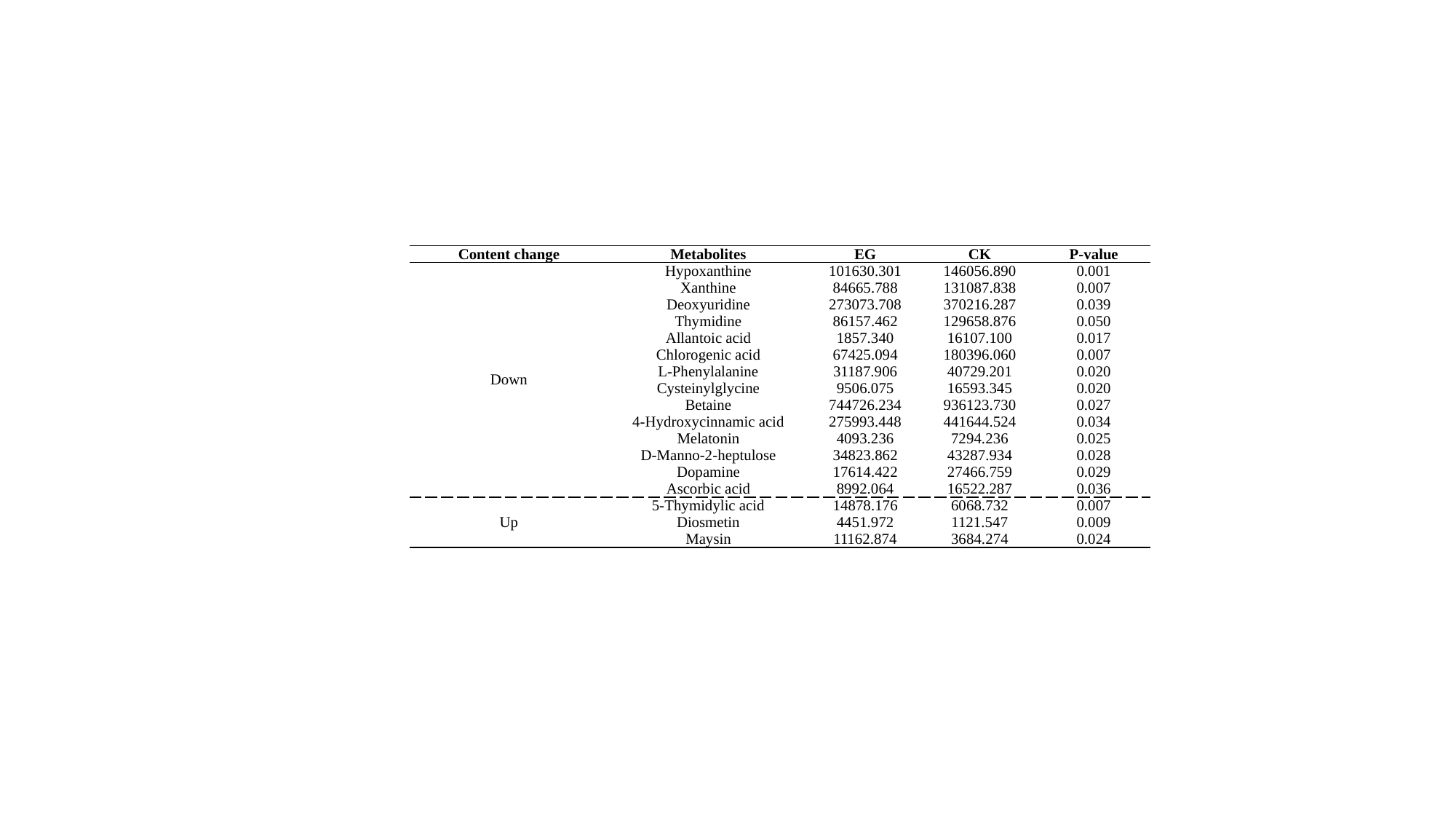

| Content change | Metabolites | EG | CK | P-value |
| --- | --- | --- | --- | --- |
| Down | Hypoxanthine | 101630.301 | 146056.890 | 0.001 |
| | Xanthine | 84665.788 | 131087.838 | 0.007 |
| | Deoxyuridine | 273073.708 | 370216.287 | 0.039 |
| | Thymidine | 86157.462 | 129658.876 | 0.050 |
| | Allantoic acid | 1857.340 | 16107.100 | 0.017 |
| | Chlorogenic acid | 67425.094 | 180396.060 | 0.007 |
| | L-Phenylalanine | 31187.906 | 40729.201 | 0.020 |
| | Cysteinylglycine | 9506.075 | 16593.345 | 0.020 |
| | Betaine | 744726.234 | 936123.730 | 0.027 |
| | 4-Hydroxycinnamic acid | 275993.448 | 441644.524 | 0.034 |
| | Melatonin | 4093.236 | 7294.236 | 0.025 |
| | D-Manno-2-heptulose | 34823.862 | 43287.934 | 0.028 |
| | Dopamine | 17614.422 | 27466.759 | 0.029 |
| | Ascorbic acid | 8992.064 | 16522.287 | 0.036 |
| Up | 5-Thymidylic acid | 14878.176 | 6068.732 | 0.007 |
| | Diosmetin | 4451.972 | 1121.547 | 0.009 |
| | Maysin | 11162.874 | 3684.274 | 0.024 |

## Slide 10
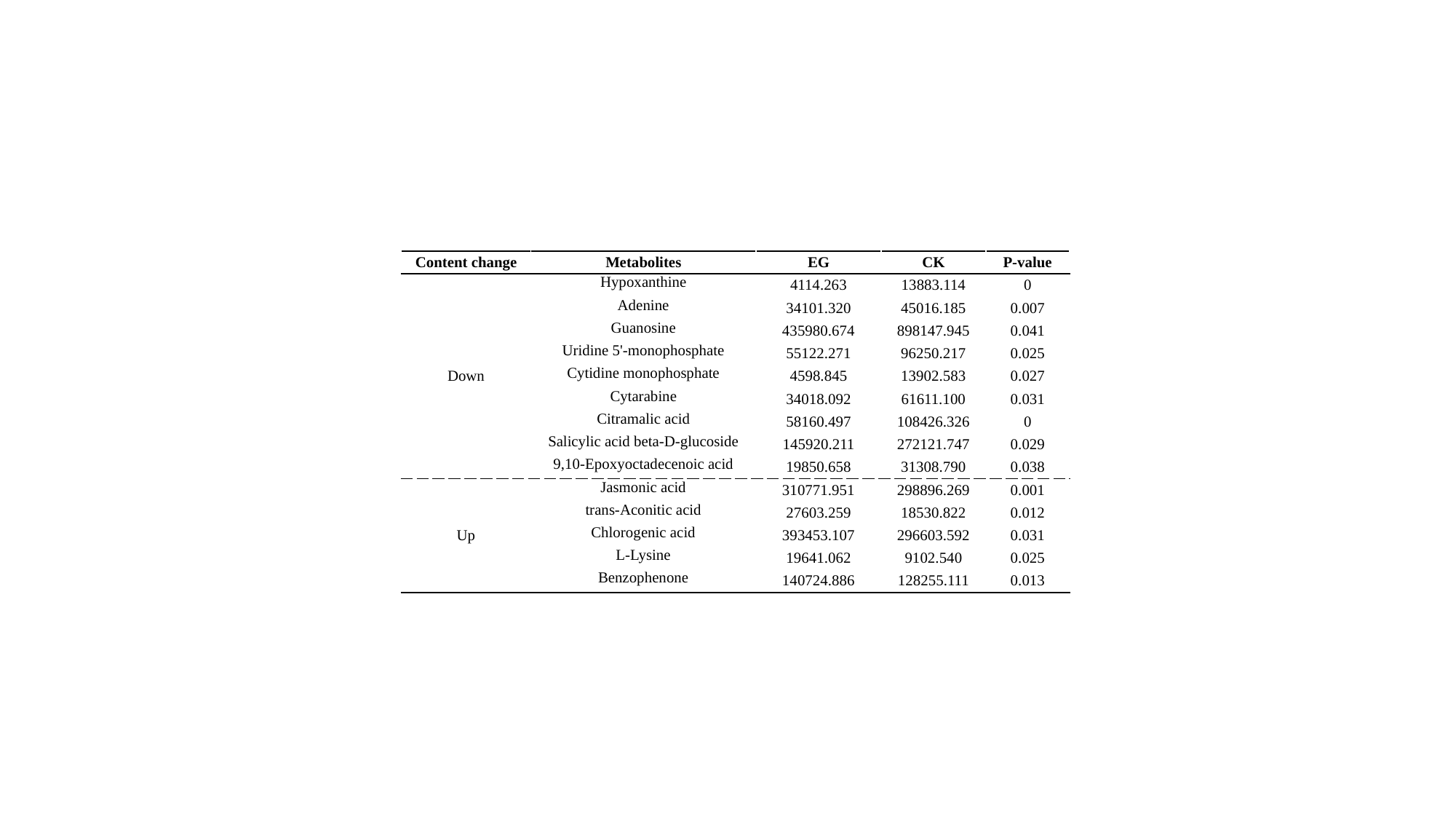

| Content change | Metabolites | EG | CK | P-value |
| --- | --- | --- | --- | --- |
| Down | Hypoxanthine | 4114.263 | 13883.114 | 0 |
| | Adenine | 34101.320 | 45016.185 | 0.007 |
| | Guanosine | 435980.674 | 898147.945 | 0.041 |
| | Uridine 5'-monophosphate | 55122.271 | 96250.217 | 0.025 |
| | Cytidine monophosphate | 4598.845 | 13902.583 | 0.027 |
| | Cytarabine | 34018.092 | 61611.100 | 0.031 |
| | Citramalic acid | 58160.497 | 108426.326 | 0 |
| | Salicylic acid beta-D-glucoside | 145920.211 | 272121.747 | 0.029 |
| | 9,10-Epoxyoctadecenoic acid | 19850.658 | 31308.790 | 0.038 |
| Up | Jasmonic acid | 310771.951 | 298896.269 | 0.001 |
| | trans-Aconitic acid | 27603.259 | 18530.822 | 0.012 |
| | Chlorogenic acid | 393453.107 | 296603.592 | 0.031 |
| | L-Lysine | 19641.062 | 9102.540 | 0.025 |
| | Benzophenone | 140724.886 | 128255.111 | 0.013 |

## Slide 11
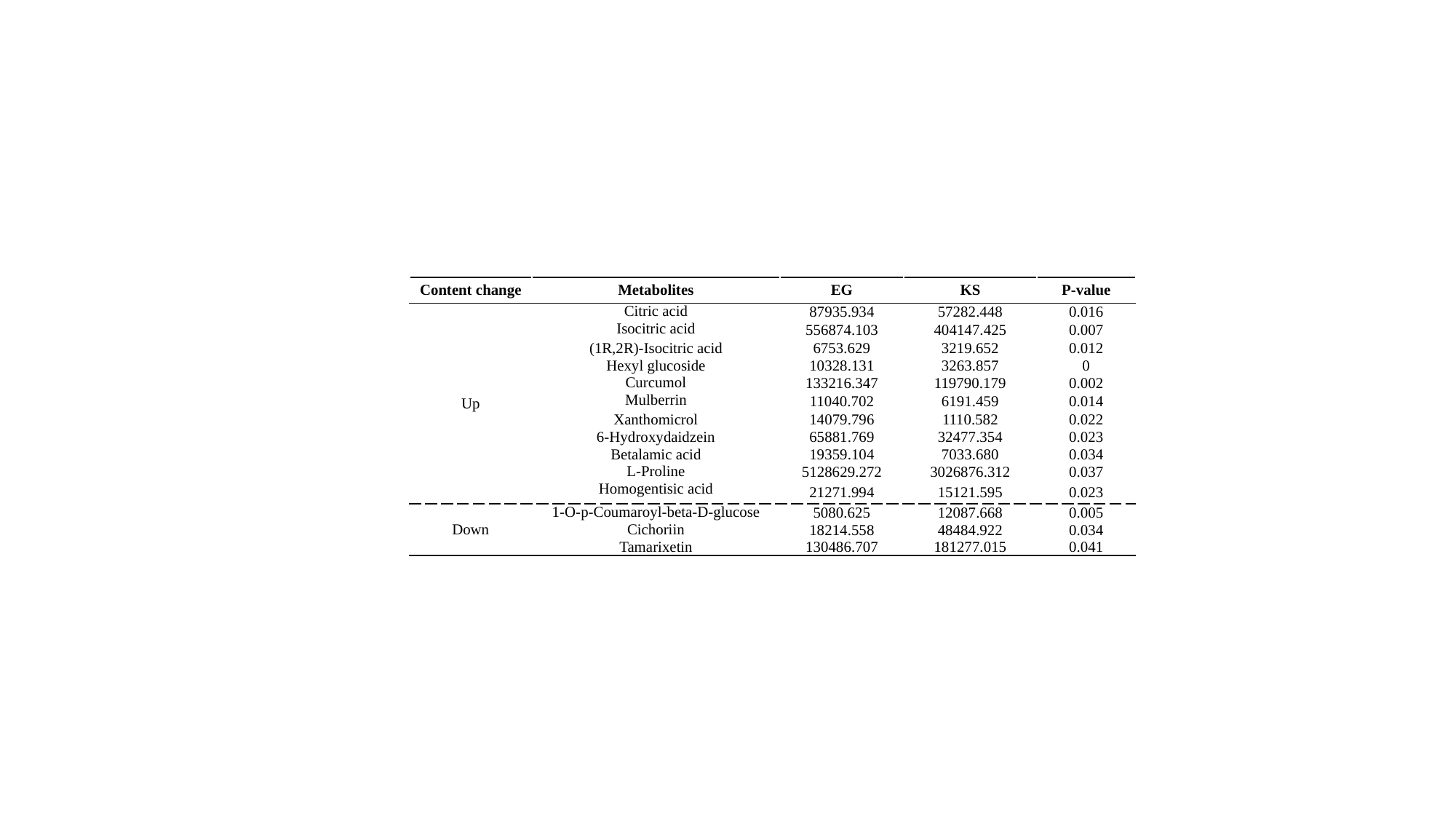

| Content change | Metabolites | EG | KS | P-value |
| --- | --- | --- | --- | --- |
| Up | Citric acid | 87935.934 | 57282.448 | 0.016 |
| | Isocitric acid | 556874.103 | 404147.425 | 0.007 |
| | (1R,2R)-Isocitric acid | 6753.629 | 3219.652 | 0.012 |
| | Hexyl glucoside | 10328.131 | 3263.857 | 0 |
| | Curcumol | 133216.347 | 119790.179 | 0.002 |
| | Mulberrin | 11040.702 | 6191.459 | 0.014 |
| | Xanthomicrol | 14079.796 | 1110.582 | 0.022 |
| | 6-Hydroxydaidzein | 65881.769 | 32477.354 | 0.023 |
| | Betalamic acid | 19359.104 | 7033.680 | 0.034 |
| | L-Proline | 5128629.272 | 3026876.312 | 0.037 |
| | Homogentisic acid | 21271.994 | 15121.595 | 0.023 |
| Down | 1-O-p-Coumaroyl-beta-D-glucose | 5080.625 | 12087.668 | 0.005 |
| | Cichoriin | 18214.558 | 48484.922 | 0.034 |
| | Tamarixetin | 130486.707 | 181277.015 | 0.041 |

## Slide 12
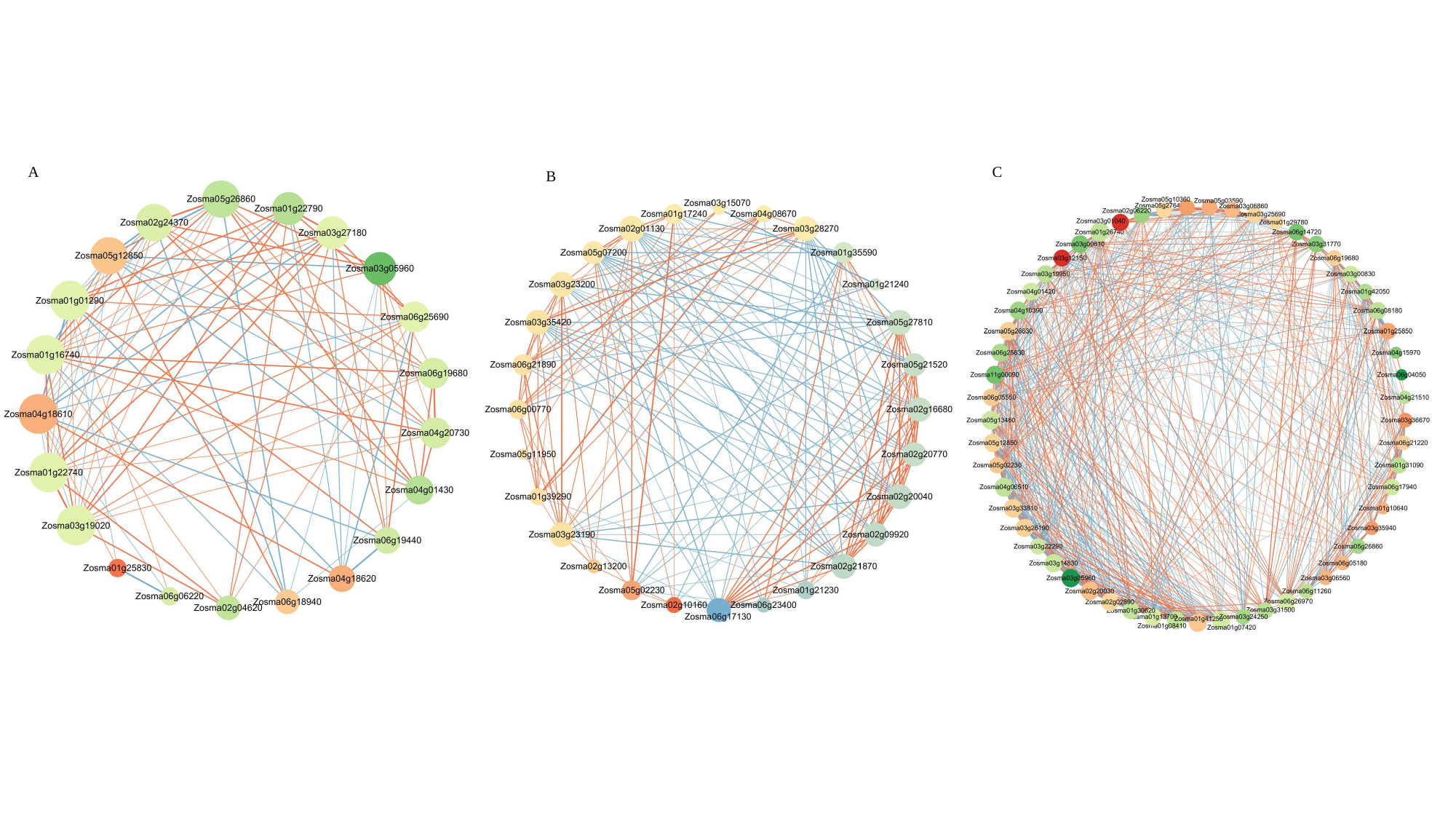

A
C
B
